# Secukinumab and Dead Sea Climatotherapy Impact Resolved Psoriasis Skin Differently Potentially Affecting Disease Memory

**DOI:** 10.3390/ijms25116086

**Published:** 2024-05-31

**Authors:** Thomas Emmanuel, Borislav Ignatov, Trine Bertelsen, Thomas Litman, Morten Muhlig Nielsen, Mikkel Bo Brent, Toke Touborg, Anders Benjamin Rønsholdt, Annita Petersen, Mette Boye, Ida Kaaber, Daniel Sortebech, Dorte Lybæk, Torben Steiniche, Anne Bregnhøj, Liv Eidsmo, Lars Iversen, Claus Johansen

**Affiliations:** 1 Department of Dermatology, Aarhus University Hospital, 8200 Aarhus, Denmark; trinbert@rm.dk (T.B.); 201709823@post.au.dk (T.T.); 201610150@post.au.dk (A.B.R.); xannita3@hotmail.com (A.P.); mettemusen21@hotmail.com (M.B.); idakaaber@hotmail.dk (I.K.); dlybaek@dadlnet.dk (D.L.); annebreg@rm.dk (A.B.); lars.iversen@clin.au.dk (L.I.); claus.johansen@clin.au.dk (C.J.); 2 Department of Clinical Medicine, Aarhus University Hospital, 8200 Aarhus, Denmark; morten.muhlig@clin.au.dk (M.M.N.); steiniche@clin.au.dk (T.S.); 3 Department of Medicine, Karolinska Universitetssjukhuset, 171 76 Stockholm, Sweden; bobbyignatov@outlook.com (B.I.); daniel.sortebech@stud.ki.se (D.S.); liv.eidsmo@sund.ku.dk (L.E.); 4 Department of Immunology and Microbiology, Copenhagen University, 2200 Copenhagen, Denmark; tlitman@sund.ku.dk; 5 Department of Molecular Medicine, Aarhus University Hospital, 8200 Aarhus, Denmark; 6 Department of Biomedicine, Aarhus University, 8000 Aarhus, Denmark; mbb@biomed.au.dk; 7 Department of Pathology, Aarhus University Hospital, 8200 Aarhus, Denmark; 8 LEO Foundation Skin Immunology Research Center, 2200 Copenhagen, Denmark

**Keywords:** inflammatory skin diseases, psoriasis, biologics, phototherapy, T lymphocytes, tissue resident memory T-cells

## Abstract

Secukinumab and Dead Sea treatment result in clear skin for many psoriasis patients, through distinct mechanisms. However, recurrence in the same areas after treatments suggests the existence of a molecular scar. We aimed to compare the molecular and genetic differences in psoriasis patients who achieved complete response from secukinumab and Dead Sea climatotherapy treatments. We performed quantitative immunohistochemical and transcriptomic analysis, in addition to digital spatial profiling of skin punch biopsies. Histologically, both treatments resulted in a normalization of the lesional skin to a level resembling nonlesional skin. Interestingly, the transcriptome was not normalized by either treatments. We revealed 479 differentially expressed genes between secukinumab and Dead Sea climatotherapy at the end of treatment, with a psoriasis panel identifying *SERPINB4*, *SERPINB13*, *IL36G*, *IL36RN*, and *AKR1B10* as upregulated in Dead Sea climatotherapy compared with secukinumab. Using digital spatial profiling, pan-RAS was observed to be differentially expressed in the microenvironment surrounding CD103^+^ cells, and IDO1 was differentially expressed in the dermis when comparing the two treatments. The differences observed between secukinumab and Dead Sea climatotherapy suggest the presence of a molecular scar, which may stem from mechanistically different pathways and potentially contribute to disease recurrence. This may be important for determining treatment response duration and disease memory.

## 1. Introduction

Psoriasis is a non-communicable chronic disease characterized by skin lesions in the form of well-delineated scaly plaques and a high comorbidity burden [1,2]. Approximately 0.1% of the world population is affected by the disease and Denmark is among the countries with the highest prevalence rates in the world at 1.8% [3]. The multi-faceted nature of the disease merits a holistic treatment approach [4,5]. Currently, many treatment options exist, ranging from topical, oral systemic, phototherapy, to various biologics [4]. Dead Sea climatotherapy (DSC) and biologic therapies, such as the anti-interleukin (IL)-17A targeting drug secukinumab (SEC), are both effective treatments for psoriasis [6,7,8]. In Denmark, government funded DSC is performed at the Dead Sea in Israel, where patients receive extensive individualized sun and saltwater treatment for four weeks. This holistic and remittive treatment results in approximately three months of continuous complete skin clearance among patients who achieve complete skin clearance i.e., psoriasis area and severity index (PASI)-100 response at end of treatment (EOT) [7]. The unique effect of DSC on psoriasis is largely attributed the region’s extremely low altitude and mud composition [9,10].

Climatotherapy utilization varies across the globe; from 8% in the Nordic countries to 46% in the Middle East [11]. In Denmark, DSC is primarily utilized for patients who have not achieved disease control with traditional treatments, or for those who have contraindications to them. It is known that psoriasis symptoms exhibit seasonal variability. Some patients experience symptom improvement during summer or with sun exposure, while others notice better outcomes during winter. Conversely, certain patients report worsening symptoms during warmer periods [12]. The molecular processes underlying how climatotherapy improves psoriasis are not well understood. Ultraviolet (UV) light inhibits Langerhans cells, alters the cytokine production in the skin, and induces T-cell apoptosis. Narrowband (NB)-UVB therapy affects immune-related genes in atopic dermatitis patients before changing skin morphology and immune cell infiltrates [13,14]. Therefore, a similar mechanism may apply in the skin during DSC.

SEC results in complete disease clearance and a PASI-100 response in 44% of patients after 16 weeks of treatment [15] and is mostly used for patients who do not achieve disease control on systemic oral medications. However, the relapsing nature of psoriasis, as demonstrated after DSC, is a challenge in psoriasis therapy. Several studies have found the presence of a “molecular scar” in resolved skin, which may be involved in disease relapse [16,17]. Pathogenic tissue-resident memory T-cells (TRMs) have been proposed as key players in disease memory in psoriasis and other chronic skin diseases [18,19,20]. TRMs are dependent on interaction with the local microenvironment, and specifically the pluripotent cytokine transforming growth factor beta (TGFbeta), the metabolic factors fatty-acid-binding proteins 4 and 5, and the cytokines IL-7 and IL-15 are involved in the differentiation and maintenance of TRMs [21,22,23,24].

With the advent of biological therapies, complete continuous skin clearance has become a realistic treatment goal for psoriasis [25,26,27,28,29], and the potential for SEC to induce long-term deep remission after early intervention is currently being investigated in the STEPIn study [30]. The STEPIn study aims to compare SEC with NB-UVB phototherapy for treating new onset plaque psoriasis in a randomized, open-label, multicenter trial. Clinical results showed that early intervention with SEC was superior to NB-UVB, and a high sustained skin clearance was observed [31]. However, what molecular signature remains in clinically resolved skin is still largely unknown. A more detailed understanding of the mechanisms inducing long-term remission, and the potential for disease memory modification, which is currently being investigated in the GUIDE study, may pave the way for more efficient treatment options with a longer treatment durability [32].

The objective of the current study was to evaluate and compare the impact of DSC and SEC treatments on the molecular scar found in clinically resolved psoriasis skin among complete responders, specifically those who achieved a complete response, i.e., PASI-100 response [6,7]. We selected these two treatments because they have distinct mechanisms of action and are highly effective in the short term for treating psoriasis. The comparison was performed using (i) quantitative immunohistochemical analysis, (ii) transcriptome analysis, and (iii) digital spatial profiling analysis (DSP).

## 2. Results

### 2.1. Dead Sea Climatotherapy and Secukinumab Treatment Reduced Epidermal Thickness, Proliferation, CD1a^+^, CD3^+^, CD4^+^, CD8^+^, CD11c^+^, CD45RO^+^, and MPO^+^ Cells

**Figure 1:**
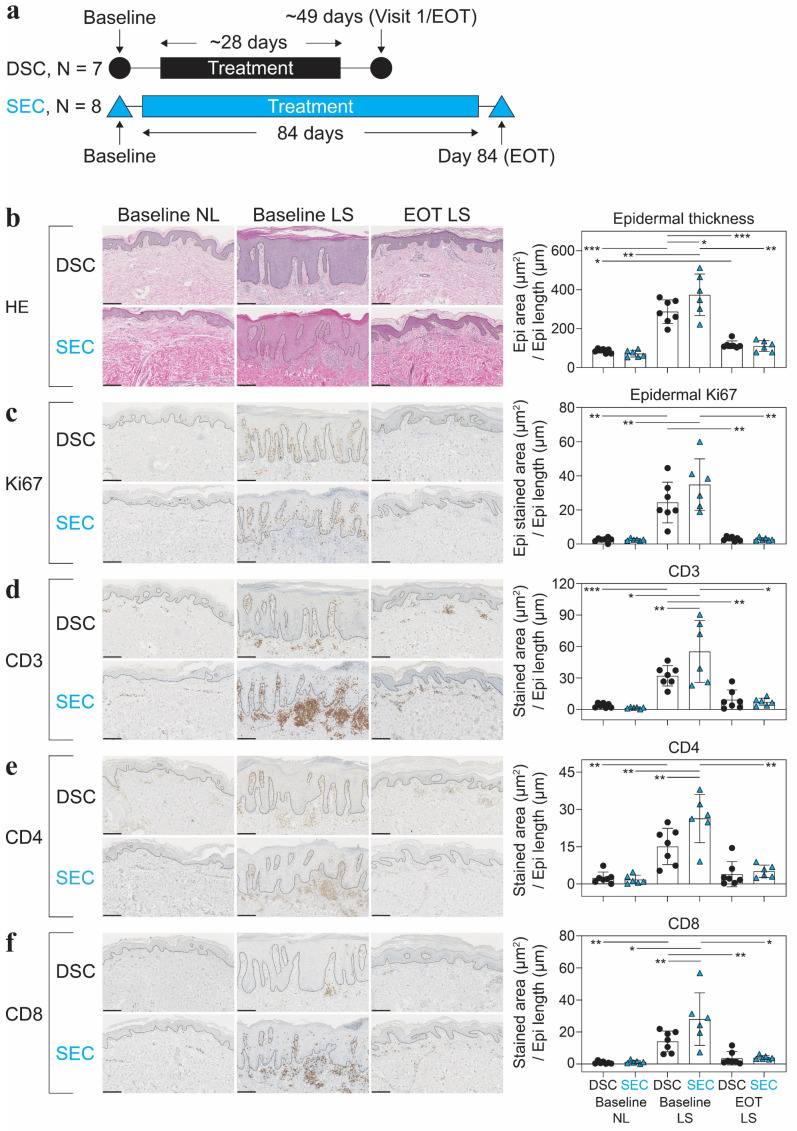
Immunohistochemical results from the two cohorts at baseline and end of treatment. (**a**) Study schematic of the treatment duration from baseline to end of treatment (EOT) for patients treated with Dead Sea climatotherapy (DSC) and patients treated with secukinumab (SEC). (**b**–**f**) Results from hematoxylin and eosin (HE) staining used to quantify the epidermal thickness and immunohistochemistry of Ki67^+^, CD3^+^, CD4^+^, and CD8^+^ cells from nonlesional (NL) and lesional (LS) skin at baseline, and LS skin at EOT. The dashed lines indicate the interface between the epidermis and dermis. Sequential slides from the same patient are shown. Scale bars = 200 μm. Mean ± SD depicted. One-way analysis of variance (ANOVA) with Tukey’s multiple comparison test was used to compare across cohorts, and repeated measures analysis of variance with post hoc Šidák test was used to compare between timepoints within studies. * *p* < 0.05, ** *p* < 0.01, *** *p* < 0.001.

Two cohorts were compared on key demographic parameters and were overall comparable (Table S1). Having established the similarity between the demographics of the two cohorts, we next measured the epidermal thickness and a selection of psoriasis-associated inflammatory biomarkers during treatment. Using hematoxylin and eosin (HE) staining, we quantified the epidermal thickness (Figure 1b).

DSC and SEC treatment both significantly reduced epidermal thickness at EOT compared with baseline LS skin. However, DSC did not completely normalize epidermal thickness to baseline NL levels. No difference between the two treatments was observed at EOT.

We also assessed the proliferation (Ki67^+^ cells) and infiltration of T-cells (CD3^+^, CD4^+^, and CD8^+^ cells) in the epidermis (Figure 1c–f). Most markers were significantly reduced when comparing baseline LS skin with EOT LS skin. The effects of both treatments were also corroborated by segmentation into an epidermal and dermal compartment (Figure S1). No difference between the two treatments was observed at EOT. We also evaluated the quantity of CD1a^+^, CD11c^+^, CD45RO^+^, and MPO^+^ cells (Figure S2). CD11c^+^ and MPO^+^ cells were significantly reduced for both DSC and SEC, while CD1a^+^ and CD45^+^ cells were only significantly reduced for the SEC group at EOT. When analyzing the dermal and epidermal compartments separately, CD1a^+^ cell quantities in the dermal compartment for the SEC-treated patients were not completely normalized to baseline NL levels (Figure S3). No difference in any of the cell quantities was observed between the two cohorts at EOT. These results indicate that both DSC and SEC treatments significantly reduced the tested histological biomarkers at EOT, with no observed difference between the two treatments in resolved skin at EOT.

### 2.2. Dead Sea Climatotherapy and Secukinumab Treatment Differentially Reduced CD15^+^, CD56^+^, CD103^+^, CD163^+^, CD207^+^, and FOXP3^+^ Cells

**Figure 2:**
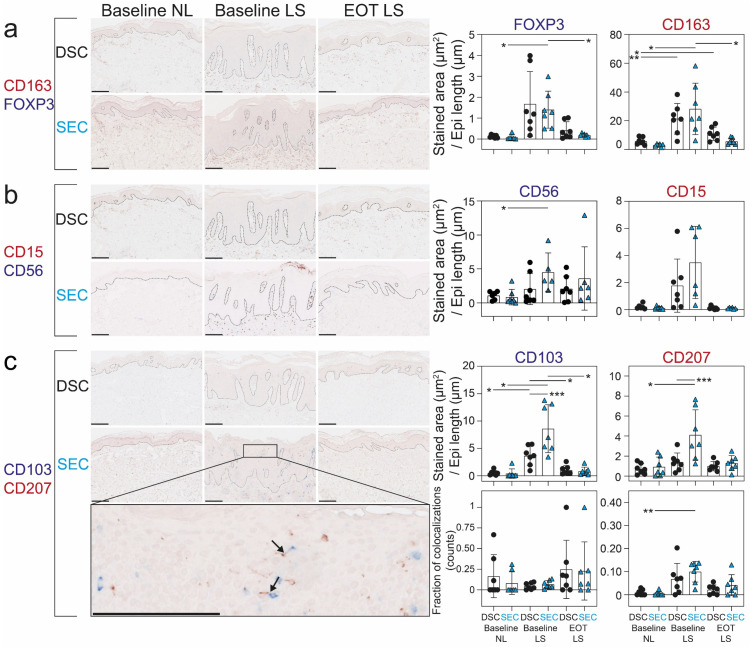
Immunohistochemical results from CD15, CD56, CD103, CD207, CD163, CD207, and FOXP3 from the two cohorts at baseline and end of treatment. (**a**–**c**) Results from quantitative immunohistochemistry analysis of CD15^+^, CD56^+^, CD103^+^, CD163^+^, CD207^+^, and FOXP3^+^ cells from nonlesional (NL) and lesional (LS) skin taken at baseline and end of treatment (EOT) from patients treated with Dead Sea climatotherapy (DSC) and patients treated with secukinumab (SEC). Black arrows show examples of CD103^+^ cells colocalizing with CD207^+^ cells. Scale bars = 200 μm. Mean ± SD depicted. One-way analysis of variance (ANOVA) with Tukey’s multiple comparison test was used to compare across cohorts, and repeated measures analysis of variance with post hoc Šidák test was used to compare between timepoints within studies. * *p* < 0.05, ** *p* < 0.01, *** *p* < 0.001.

To further elucidate if differences existed in other psoriasis-related biomarkers within the skin, we performed quantitation of additional immune cells known to be involved in the pathogenesis of psoriasis (Figure 2).

Treatment with SEC reduced the number of FOXP3^+^, CD163^+^, and CD103^+^ cells from baseline to EOT, while DSC only reduced CD103^+^ cells. In the DSC group, a significant amount of CD163^+^ cells were still present in clinically healed skin. In general, similar results were found when analyzing the dermal and epidermal compartments separately (Figure S4). We also assessed direct contact between CD207^+^ cells and CD103^+^ cells in the epidermis as a proxy of antigen presentation. The fraction of CD207^+^ cells co-localizing with CD103^+^ cells was increased in baseline LS skin compared to baseline NL skin (Figure 2c). No difference between treatment groups was observed at EOT.

### 2.3. CD8^+^CD49a^+^ T-Cell Counts Correlated with Disease Severity But the Proportion of CD8^+^CD49a^+^ T-Cells Out of CD8^+^ and CD49^+^ T-Cells Did Not Change during Treatment

**Figure 3:**
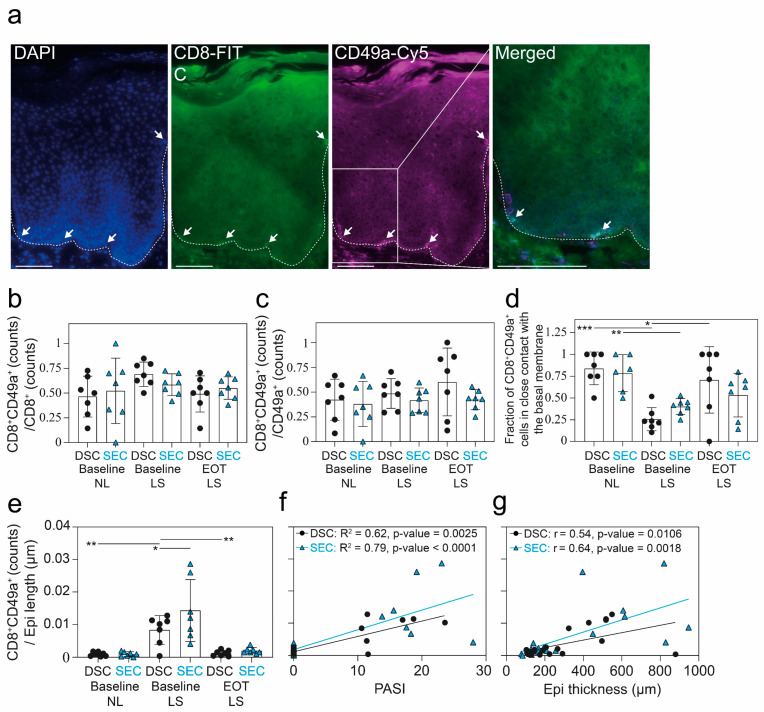
Immunohistochemical results from CD8^+^ and CD49a^+^ staining. (**a**) Representative CD8 and CD49a staining from lesional skin (LS) showing the typical localization of tissue-resident memory T-cells near the epidermal dermal border. White arrows indicate examples of CD8^+^CD49a^+^ cells. The dashed line indicates the interface between epidermis and dermis. Scale bar = 100 μm. (**b**–**d**) Results from quantitative immunohistochemistry analysis of CD8^+/−^CD49a^+/−^ cells in the epidermis from baseline nonlesional (NL), LS, and end of treatment (EOT) LS skin. (**e**) CD8^+^CD49a^+^ cell counts normalized to epidermal length from DSC and SEC treatments at baseline and EOT. (**f**) Spearman correlation for PASI and CD8^+^CD49a^+^ cell counts for DSC and SEC treatments. (**g**) Pearson correlation for epidermal thickness and CD8^+^CD49a^+^ cell counts for DSC and SEC treatments. For figure (**b**–**e**), one-way analysis of variance (ANOVA) with Tukey’s multiple comparison test was used to compare across cohorts, and a mixed model analysis with post hoc Šidák test was used to compare between timepoints within studies. Mean ± SD depicted. * *p* < 0.05, ** *p* < 0.01, *** *p* < 0.001.

CD49a expression functionally delineates distinct subsets of TRMs, and in psoriasis, CD49a^+^ TRMs have the capacity to co-produce IFNγ and IL-17 [33]. Moreover, inhibition of CD49a stopped development of psoriasis in a model where non-lesional skin was transplanted to immunodeficient mice [34]. Thus, we investigated whether CD8^+^CD49a^+^ cell counts changed during treatment. The vast majority of CD8^+^CD49a^+^ cells were found in close contact with the basal membrane in NL and EOT LS skin (Figure 3a), and this number was significantly reduced in LS skin compared with NL skin (Figure 3d).

No significant difference could be observed in the proportion of the CD8^+^CD49a^+^ double-positive population out of the CD8^+^ T-cells or CD49a^+^ cells across the timepoints (Figure 3b,c). Psoriasis lesions were particularly enriched with CD8^+^CD49a^+^ T-cells; however, no difference between groups was observed at EOT (Figure 3e). In addition, a clear correlation was observed between CD8^+^CD49a^+^ T-cell counts and both epidermal thickness and PASI (Figure 3f,g).

### 2.4. Dead Sea Climatotherapy and Secukinumab Treatment Altered the Transcriptome in Patients Responding to Treatment

After conducting a thorough histological analysis and delineating the cellular profile in lesional psoriatic skin and in clinically resolved psoriatic skin after SEC or DSC treatments, we next wanted to investigate and compare the molecular alterations following these treatments. To this end, we used Clariom D microarrays covering more than 542,000 transcripts from baseline NL, baseline LS, and EOT LS skin. The profound molecular response to both treatments is illustrated in the heatmap and semi-supervised clustering (Figure S5a–c). As expected, a large difference between the gene expression profile of baseline NL and LS skin was observed, with 2751 DEGs for the combined cohorts (Figure S6a). Many DEGs in LS skin were typical psoriasis signature genes (Figure S6b–d). The S100 family signaling pathway was significantly activated in psoriasis skin, supporting a psoriasis-specific gene signature (Figure S7). Several known psoriasis-related genes were also differentially expressed for each cohort separately, suggesting a high degree of homogeneity between the two cohorts at baseline (Figure S8).

### 2.5. A Molecular Scar Was Still Present in Completely Resolved Psoriasis Skin after Dead Sea Climatotherapy and Secukinumab Treatment

Resolved psoriasis skin contains a transcriptome that differs from NL skin [35,36]. To investigate this disease memory and to determine differences between the two treatment modalities, we compared the baseline NL skin with EOT LS skin in clinically resolved psoriasis skin in the two cohorts (Figure S9). In the DSC cohort, a clear separation of the two timepoints was observed. As expected, among the top-25 upregulated DEGs, many were involved in melanogenesis (Figure S9a,b). The SEC cohort was also clearly divided according to the two timepoints (Figure S9c,d). Again, pathway analysis for both groups suggested a residual psoriasis signature and IL-17A signaling in fibroblasts (Figures S10 and S11). The psoriasis-related DEGs in EOT LS compared with NL skin from the two treatments confirmed the existence of a disease memory or “molecular scar” in visually resolved psoriatic skin.

### 2.6. Dead Sea Climatotherapy and Secukinumab Treatment Differentially Altered the Transcriptome in Clinically Resolved Psoriasis Skin

**Figure 4:**
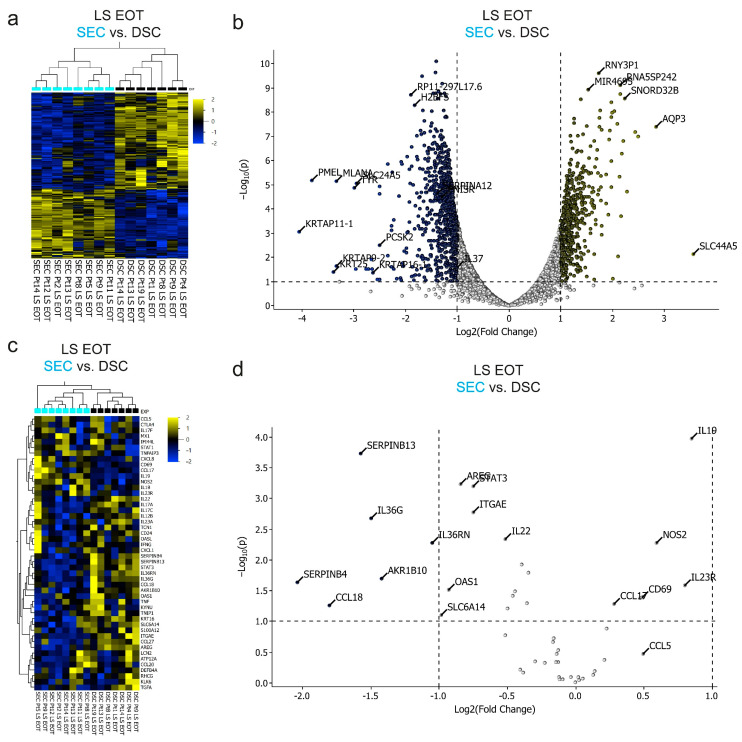
Microarray analysis of resolved skin from the two treatment cohorts. (**a**) Heatmap and two-way semi-supervised clustering based on 1217 DEGs between the SEC and DSC groups at end of treatment (Var > 0.1, *p* < 0.05, *q* = 0.10). The samples are colored according to study: DSC, black; SEC, cyan. The colors in the heatmap signify high (yellow) or low (blue) expression of a particular gene across samples (z-scaled values). (**c**) Heatmap and two-way semi-supervised clustering based on 48 psoriasis-defining genes. The samples are colored according to study: DSC, black; SEC, cyan. The colors in the heatmap signify high (yellow) or low (blue) expression of a particular gene across samples (z-scaled values). (**b**) Volcano plot showing the log2 (fold change) between the SEC and DSC groups at end of treatment for all genes on the *x*-axis and the −log10 of the *p*-value for the two groups’ (SEC vs. DSC) *t*-test on the *y*-axis. The genes that met the cut-off criteria (*p* < 0.05 and >2-fold change) are colored in the same way as for the heatmap (higher/lower in SEC, yellow/blue). (**d**) Heatmap and semi-supervised hierarchical clustering showing the six most significantly different upregulated and downregulated DEGs based on the 48 psoriasis-related genes.

To better understand the molecular environment in resolved skin, we performed a comparison of the transcriptome between the DSC and SEC cohorts, with a focus on EOT LS skin. The heatmap and semi-supervised hierarchical clustering based on 479 DEGs between DSC and SEC at EOT illustrated a clear difference between the two cohorts (Figure 4a,b).

Among the top DEGs, several were pseudogenes and noncoding genes (e.g., *RNY3P1* and *SNORD32B*), while others were related to the sun treatment (e.g., *PMEL* and *MLANA*). Some DEGs, however, are known to be involved in the pathogenesis of psoriasis (e.g., *SERPINB3* and *SERPINB4*) [37,38]. To better illustrate the differences between the effect of DSC and SEC treatments, we performed semi-supervised hierarchical clustering of samples based on a panel consisting of 48 psoriasis-defining genes selected from a range of studies that compared the bulk transcriptome in NL skin with LS skin, in addition to some involved in TRM cell biology [39,40,41,42,43,44,45] (Table S5) (Figure 4c,d). The role of some of these genes in psoriasis has not been fully elucidated. Based on this panel, five DEGs were identified as upregulated in DSC compared with SEC (*SERPINB13*, *IL36G*, *IL36RN*, *SERPINB4*, and *AKR1B10*) (Figure 4d).

In summary, these results suggest that the two treatments altered the psoriasis-transcriptome differentially in resolved skin at EOT.

### 2.7. Digital Spatial Profiling Successfully Assessed the Proteome of CD45^+^ Cells, MelanA^+^ Cells, and the Microenvironment in the Skin

**Figure 5:**
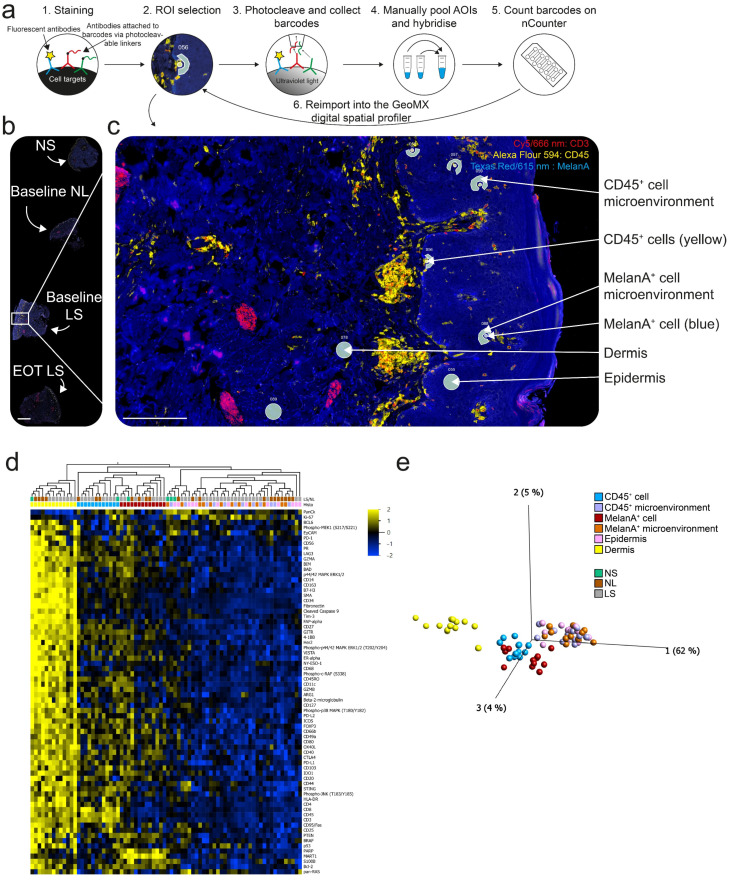
Schematic of the digital spatial profiler workflow and assessment of CD45^+^ cells, MelanA^+^ cells, and the microenvironment in the skin. (**a**) (1) Slides were incubated with a cocktail of primary labeled fluorescent antibodies mixed with a panel of commercial NanoString antibodies linked to photocleavable barcoded oligos. (2) Areas of illumination (AOIs) were selected in the digital spatial profiler (DSP) using fluorescent morphology markers to distinguish tissues and cells. (3) Ultraviolet light separated the oligos from the antibodies. The oligos were then pulled by a microcapillary tip and deposited in individual wells on a 96-well plate. (4) Cells were manually pooled from several wells into one well and hybridized according to the NanoString protocol. (5) Oligos were counted in the nCounter and the results could then be imported into the DSP, allowing real-time spatial analysis of individual AOIs and normalizations of values. Real-time spatial analysis was limited in this experiment, due to the pooling of AOIs. (**b**) Image showing the setup on the slides with normal skin (NS), baseline nonlesional (NL), baseline lesional (LS), and EOT LS. Size bar = 1 mm. (**c**) Larger image of baseline LS showing the ROIs selected. Three 50 μm diameter ROIs from the dermis and epidermis were collected from a random area without visible CD3^+^, CD45^+^, or MelanA^+^ cells. Ten 10 μm diameter ROIs from CD45^+^ cells (yellow) and MelanA^+^ cells (blue) were collected from the epidermis and ten 10–20 μm width contour sections surrounding the CD45^+^ cells (CD45^+^ cell microenvironment) and MelanA^+^ cells (MelanA^+^ cell microenvironment) were selected using the polygon tool. Scale bar = 200 μm. (**d**) Heatmap and two-way unsupervised clustering based on the 70 most variable proteins (Var > 0.2) across samples. The samples are colored according to tissue (healthy skin, green; NL, brown; LS, gray) and histology. The colors in the heatmap signify high (yellow) or low (blue) expression of a particular gene across samples (z-scaled values). The samples clustered primarily according to dermis, but also according to CD45^+^ and MelanA^+^ cells. (**e**) PCA plot based on the same 70 proteins, colored according to histology.

Locally expressed disease-related genes in focal regions of psoriasis vulgaris skin lesions are known to drive microenvironmental and cellular changes [46]. Melanocytic autoantigens have been proposed to trigger T-cell production of IL-17 and IFN-γ [47], and we thus wanted to investigate the microenvironment surrounding both immune cells (CD45^+^ cells) and melanocytes (MelanA^+^ cells). We thus applied digital spatial profiling (DSP) using the NanoString GeoMX platform in two SEC patients and two DSC patients. DSP allows for the non-destructive investigation of protein or RNA expression in selected areas of illumination (AOIs) from formalin-fixed paraffin-embedded (FFPE) skin tissue sections [48]. Currently, the smallest resolution of the DSP is 10 μm diameter circles [49]. We pooled CD45^+^ cells, the surrounding CD45^+^ cell microenvironment, MelanA^+^ cells, the surrounding MelanA^+^ cell microenvironment, 50 μm diameter dermal, and 50 μm diameter epidermal areas from baseline NL, baseline LS, EOT LS skin from two DSC and two SEC patients, and one normal skin control (Figure 5a–c). The method has recently been published [50].

The full list of modules used is presented on Table S6. Clustering of the dermis, CD45^+^ cells, and MelanA^+^ cells is illustrated in the heatmap and PCA plot (Figure 5d,e). However, clustering according to timepoints or treatments was not observed.

### 2.8. Digital Spatial Profiling of the Proteome in Selected Areas of the Skin Showed Distinct Differences in Resolved Skin from Dead Sea Climatotherapy and Secukinumab Treatment

We next sought to further investigate the microenvironment in the skin through DSP.

Because the transcriptome was differentially regulated at EOT, we also compared the proteome at focal areas in the skin at EOT between SEC and DSC treatments. We selected areas of illumination (AOIs) consisting of CD103^+^ cells by segmentation (Figure S12a,b), the epidermis (Figure S12b, red area), the microenvironment (10–20 μm) surrounding one or more CD103^+^ cells (Figure S12c), a dermal infiltrate (Figure S12d), and the top of a dermal papilla (Figure S12e). CD103^+^ cells and the top of a dermal papilla were only acquired from baseline SEC LS samples. Comparison between baseline NL, baseline LS, and EOT LS showed clustering of the samples according to cell type and AOI (Figure S13).

**Figure 6:**
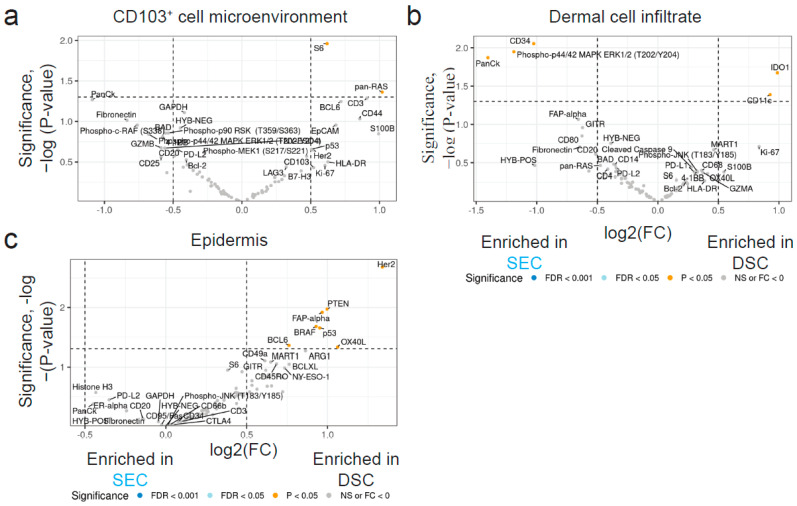
Differentially expressed proteins at end of treatment between the two cohorts. (**a**) Differentially expressed proteins (DEPs) in the CD103^+^ microenvironment between Dead Sea climatotherapy (DSC) and secukinumab (SEC)-treated patients at end of treatment (EOT). (**b**) DEPs in the dermal infiltrate between DSC and SEC-treated patients at EOT. (**c**) DEPs in the epidermis between DSC- and SEC-treated patients at EOT.

When comparing EOT samples, we found several differentially expressed proteins (DEPs) (Figure 6).

panRAS was significantly upregulated in the CD103^+^ cell microenvironment in DSC compared with SEC skin (Figure 6a). CD34, P-p44/42, and PanCK were downregulated in DSC, and IDO1 and CD11c were upregulated in DSC, compared with SEC in the dermal infiltrate at EOT (Figure 6b). In the epidermis BCL6, OX40L, p53, BRAF, FAP-alpha, PTEN, and HER2 were upregulated at EOT in DSC compared with SEC patients (Figure 6c). In addition, we also compared AOIs between baseline NL and EOT LS skin and found several DEPs (Figure S14). In summary, the results suggest that the two treatments differentially altered the proteome in focal areas.

## 3. Discussion

The present study aimed to investigate whether any cellular or molecular differences existed in resolved skin at EOT between DSC and SEC among patients that were complete responders. Interestingly, the treatments differentially impacted the transcriptome and proteome at EOT. It is well established that the residual transcriptomic profile is not fully resolved in macroscopically cleared psoriasis skin [51]. In line with this, the epidermal thickness was still significantly increased in DSC-treated skin at EOT. Changes in immune cell infiltrates and the transcriptome precede morphological changes in epidermal thickness, so this observation might not only signify a delay in treatment response but also UVB-induced hyperproliferation of the epidermis [52,53,54]. Furthermore, immunohistochemistry showed more CD1a^+^ cells in the dermis of resolved skin after SEC treatment. CD1a^+^ cells play an important role in antigen presentation in psoriasis, but further research into their role is needed [55]. Several studies investigated the histological presence of immune cells after 12 weeks of treatment and observed that LS skin resembles NL skin by week 12 of SEC treatment and TNF inhibition [56,57]. In general, in our study, the SEC-treated cohort had higher baseline quantities of immune cells in LS skin compared with the DSC-treated cohort. One limitation was the low number of patients and the large difference in cell quantity seen in resolved skin among patients. Transcriptomic profiling has provided important insights into disease biology, disease mechanism, and discovery of important biomarkers for therapeutic response [58,59,60]. Both treatments normalized the LS transcriptomic profile, associated with aberrant keratinocyte biology in psoriatic skin, almost leading to molecular resolution, in line with previous reports [56,61]. We also confirmed that resolved skin still harbored a psoriasis-related transcriptome [36]. Differences between the two cohorts at EOT included psoriasis-related genes *SERPINB4* and *SERPINB13*, which belong to a group of keratinocyte differentiation regulators, *IL36G* and *IL36RN*, which are biomarkers for psoriasis lesions and correlate with disease activity [62,63], and *AKR1B10* which plays an important role in keratinocyte proliferation [64]. The DEGs found at EOT when comparing the two cohorts were thus mostly related to keratinocyte differentiation and proliferation. Interestingly, the IL-17A pathway in fibroblasts was still significantly upregulated in the resolved skin. Fascinatingly, the onset of normalization of LS skin from DSC was rapid and may currently be the most effective short-term treatment for psoriasis. The essential role of the IL-17 and IL-23 pathway in psoriasis was underscored by the efficacy of IL-17 inhibitors such as SEC in the treatment of psoriasis [65,66,67]. Many patients with moderate-to-severe psoriasis initiating systemic treatment have a high likelihood of achieving almost full skin-clearance after initiating therapy. In addition, the efficacy of newer drugs has allowed for the term “complete responders” to designate patients who achieve complete or near complete skin clearance [68]. A subanalysis of lesional skin biopsy specimens from the ECLIPSE study showed reduced frequencies of CD8^+^ TRMs from guselkumab (an IL-23 inhibitor) treatment compared with SEC treatment. SEC also decreased the frequency of T regulatory cells, whereas the frequency of T regulatory cells was maintained in biopsies of patients receiving guselkumab [69]. The differential effects of the two biological treatments may influence the resolved skin by regulating genes involved in the survival of TRMs. Moreover, pathogenic TRMs seem to persist at sites of clinically resolved psoriatic lesions, and it is unclear whether IL-23 inhibition is sufficient to modulate these cells [70]. Even after long-term therapy, TRMs do not lose their ability to produce IL-17A [17,33]. RUNX2 and RUNX3 are known to promote the differentiation of cytotoxic TRMs; however, it is currently not known to what extent modulation of these transcription factors is achieved using newer biologics [71]. Pathogenic TRMs play an important role in psoriasis and may stay in the skin for at least 10 years [72]. Targeting skin TRMs or using TRMs as an index for disease severity and deep remission has been proposed in psoriasis [73]. The interaction between T-cells trafficking into inflamed tissues and endothelial cells may prime residency and long-term chronic inflammation [74]. A correlation between the expression of TRM markers in the epidermis from plaque psoriasis lesions and the duration of skin lesions has been described [75,76]. Using correlation analysis between epidermal thickness and cell counts, we confirmed this observation. In addition, the correlation between PASI and cell counts was observed to be consistent with results performed on CD8^+^CD103^+^CD49a^+^ T-cells in psoriasis [77]. Using DSP, we examined the TRMs as defined by the CD103^+^ tissue-residency marker. Though only pan-RAS was found to be differentially regulated in the microenvironment around CD103^+^ cells at EOT between samples, we observed significant upregulation of IDO1 in the dermis of DSC-treated patients. IDO1 has been shown to induce regulatory T-cell development and inhibit T-cell activation in vitro [78]. This suggests that focal and general changes in the skin may help distinguish resolved skin from various treatments. The pre-selected protein panels used in this study only included a few proteins relative to the whole transcriptome, and future techniques such as CosMX may enable the detection of a larger quantity of proteins and with higher resolution [79]. The results presented here represent a step toward understanding the therapeutic mechanisms of DSC and SEC treatment on psoriatic skin and underscore that resolved skin still retains a signature—a molecular scar—separating it from NL skin [37]. Both treatments accordingly led to distinct EOT “molecular scars”, despite the clinical and histological similarity. The jury is still out on whether the promising concept of “disease modification” in psoriasis, caused by long-term effective therapy combined with early intervention, may change the course of psoriasis by preventing an inflammatory disease memory [80,81]. A recent study showed that patients with short disease duration had a longer time to relapse after stopping SEC treatment, substantiating the benefit of early intervention [82]. However, further mechanistic studies are needed, to investigate the significance of the DEGs and DEPs discovered in clinically resolved skin, particularly their role in the memory of inflammatory diseases such as psoriasis. In conclusion, we found that resolved skin at EOT between DSC and SEC differed in the transcriptome and proteome. This might be important for understanding the molecular scar and disease memory seen in psoriasis.

## 4. Materials and Methods

### 4.1. Study Populations

A schematic diagram of the study intervention and treatment duration can be seen in Figure 1a. In summary, 7 patients from a DSC cohort study, and 8 patients from a SEC cohort study who achieved PASI-100 response at end of treatment (EOT) were selected and used for the subsequent analysis [6,8]. All patients self-identified as white. Both studies were conducted in compliance with the Declaration of Helsinki, and signed informed consent was obtained from each patient prior to inclusion in the studies (permission number: m-20090102).

### 4.2. Statistical Analyses and Methods

Values are presented as mean ± SD and shown as individual values whenever possible. Figures and statistical analysis for immunohistochemistry were performed in GraphPad Prism (RRID:SCR_002798, v9.3.0, GraphPad Software, Inc., San Diego, CA, USA). All images were compiled in Adobe Illustrator or Adobe Photoshop (RRID:SCR_010279 and RRID:SCR_014199, Adobe Inc., San Jose, CA, USA). Missing data were not included in the statistical analysis. The Transcriptome Analysis Console 4.0 software (RRID:SCR_016519, Thermo Fisher Scientific, Waltham, MA, USA) was used to analyze transcriptome data. Differentially expressed genes (DEGs) were identified by ANOVA (cut-off: 2-fold change and *p* < 0.05), and the significance was adjusted for multiple testing by estimating false discovery rates (FDR). Data were visualized in Qlucore Omics Explorer v. 3.9 (Qlucore AB, Lund, Sweden). Volcano plots were used for interpreting differential gene and protein expression results. A *p*-value of less than 0.05 was considered significant. Additional analyses and methods are found in the Supplemental Materials (Tables S2–S4).

## Data Availability

Datasets related to this article can be found at (https://www.ncbi.nlm.nih.gov/geo/query/acc.cgi?acc=GSE137218, accessed on 1 May 2024), hosted at GEO repository.

## Supplementary Materials

The following supporting information can be downloaded at: https://www.mdpi.com/article/10.3390/ijms25116086/s1. Refs. [50,83,84] are cited in the Supplemental Materials file.
