# Supplementary material for: Secukinumab and Dead Sea Climatotherapy Impact Resolved Psoriasis Skin Differently Potentially Affecting Disease Memory
Source: Int J Mol Sci. 2024 May 31;25(11):6086. doi: 10.3390/ijms25116086 (PMC11172747; doi:10.3390/ijms25116086)
Supplement: Supplementary file 1 [file ijms-25-06086-s001.zip › ijms-3017921-supplementary.pdf]

Supplemental Materials

<u>**Supplemental Materials and Methods.**</u>

Statistics

The Shapiro–Wilk test and normal Q-Q Plots were used to analyze for normal distribution. One-way analysis of variance (ANOVA) with Tukey's multiple comparison test was used to compare across cohorts and repeated measures analysis of variance with the post hoc Šidák test was used to compare between timepoints within studies. For non-parametric data, a Friedman repeated measures analysis of variance on ranks was performed.

Biopsies and Immunohistochemistry

Three or four-mm full thickness skin punch biopsies were acquired from NL and LS skin. Biopsies were formalin-fixed and paraffin-embedded (FFPE). Resolved skin was clinically defined as skin lacking typical signs of psoriasis such as scaling and macroscopic inflammation i.e., PASI 0. For immunohistochemistry analysis, 4 micron thick FFPE tissues were deparaffinized, rehydrated and heated in TEG buffer (TRIS [10mM] 1.21 g/L, EGTA [0.5mM] 0.19 g/L, pH = 9.0) for antigen unmasking. We subsequently utilized the protocol as recommended by the manufacturer. Markers used are seen in **Table S2.** Due to exhaustion of FFPE skin biopsies by prior utilization, it was not possible to stain all patients at timepoints with all the markers. Biopsy sites are listed on **Table S3**.

Immunohistochemical Analysis

All slides were digitized for image analysis using the whole slide digital pathology scanner NanoZoomer 2.0-HT (RRID:SCR_021658, Hamamatsu Photonics K.K, Hamamatsu City, Japan) with a 20× objective. Quantitative image analysis for epidermal thickness, CD3, CD4, CD8, CD11c, and MPO was performed with the artificial intelligence (AI) assisted

application in VIS Image Analysis Software v. 2019.12.0.6957 (RRID:SCR_021711, Hørsholm, Denmark). For CD1a, CD15, CD56, CD45RO, CD103, CD163, CD207, and FOXP3 the pixel classification tool in the open-source software QuPath (RRID:SCR_018257, version 0.2.3)[1] was used using methods as previously explained [2]. The dermal area was automatically defined as 400 μm beneath the ventral epidermal surface. Immunofluorescence images were acquired in LAS AF (Leica Microsystems, Wetzlar, Germany, v. 2.6.0.7266) using a Leica DFC310 FX and with a 20× objective. Cell concentrations for $CD8^{+}CD103^{+}CD49a^{+}$ T-cells were performed in Photoshop and reported as stained area or counts divided by the epidermal length. Appropriate isotype controls were used.

RNA purification

Snap frozen punch biopsies for RNA isolation were transferred to 1 ml of –80°C cold RNAlater-ICE (Thermo Fisher Scientific, Waltham, MA). Twenty-four hours before RNA purification samples were transferred to –20°C. Upon RNA purification biopsies were removed from RNAlater-ICE, and transferred to SV RNA lysis buffer (Promega, Madison, WI) and homogenized. RNA was then isolated according to the manufacturer's instructions (Promega). The amount of RNA in each sample was measured using the Nanodrop 2000 (RRID:SCR_020309, Thermo Fisher Scientific). The RNA quality was assessed using a Bioanalyzer 2100 (RRID:SCR_019715, Agilent Technologies, Waldbronn, Germany).

Global Gene Expression Analysis

100 ng RNA from the collected biopsies were hybridized to Affymetrix Gene Chip Affymetrix Clariom D (Affymetrix, Santa Clara, CA) by Eurofins, Aarhus, Denmark. The microarray data were summarized by applying the SST-RMA (signal space transformation – robust multi-array average) gene level method as implemented in the Transcriptome Analysis

Console (TAC) 4.0 software (RRID:SCR_016519, Thermo Fisher Scientific). Differentially expressed genes (DEG) were identified by ANOVA (cut-off: 2-fold change and $P < 0.05$) and significance was adjusted for multiple testing by estimating false discovery rates (FDR). Data were visualized in Qlucore Omics Explorer v. 3.9 (Qlucore AB, Lund, Sweden), including principal component analysis (PCA), volcano plots heatmaps, and semi- and unsupervised hierarchical clustering.

Functional analysis, including pathway, upstream regulator, and network analysis, was performed in Ingenuity Pathway Analysis (RRID:SCR_008653, IPA, Qiagen, Redwood City, CA). Particularly, for the upstream regulator analysis, the Ingenuity Knowledge Base was queried for both overlap (evaluated by Fisher's Exact Test, where the p-value indicates the significance of the overlap between the differentially expressed genes and the genes predicted to be regulated) and directionality of the interaction; that is, whether the observed direction of gene expression change was mostly consistent with an activation or inhibition state of a given upstream regulator. The latter is here defined as any molecule – including transcription factors, cytokines, microRNAs, and chemical compounds - that can affect the expression of other molecules. The predicted activation state is reported by the activation z-score, which is positive in case of activation and negative in case of inhibition. The expression data are deposited in Gene Expression Omnibus. Baseline NL and baseline LS from SEC patient 12 were not included in all comparisons due to being outliers caused by contamination with methicillin-resistant *Staphylococcus aureus.*

Digital Spatial Profiling

Using NanoStrings GeoMX digital spatial profiler (DSP) (GeoMx Digital Spatial Profiler, RRID:SCR_02166, NanoString Technologies, Seattle, WA, USA) we performed multiplexed and spatially resolved profiling analysis on FFPE skin tissue samples. Five-µm thick FFPE

slides were stained with fluorescently conjugated antibodies to CD3, CD8, CD103, MelanA, CD45, and SYTO13 (see **Table S4**) according to the manufactures protocol combined with the packages and spike-ins listed in **Table S5**. A schematic of the workflow is seen on **Figure 6** and is based on a recently published method [3]. Following 20x fluorescence scanning to obtain a high-resolution image of the tissue, areas of illumination (AOIs) were selected. In the first experiment, ten-µm diameter circles were selected from ten $CD45^{+}$, and $MelanA^{+}$ cells and three 50-µm diameter circles from both the epidermis and dermis. This almost allowed for single cell resolution in a spatial context. Furthermore, a contour segment surrounding each $CD45^{+}$, and $MelanA^{+}$ cells of approximately ten-µm width was manually made using the polygon tool. In total up to 46 AOIs were selected from each biopsy. Oligos were then manually pooled before being quantified on the nCounter (NanoString nCounter Analysis System, RRID:SCR_021712, NanoString Technologies, Seattle, WA, USA). For analysis, digital counts were first normalized with internal spike-in controls (External RNA Control Consortium; ERCCs) to account for technical variation and normalized to the combined geometric mean of the three housekeeper genes (histone H3, S6 and GAPDH). Counts with a signal-to-noise ratio <2 were excluded.

SUPPLEMENTAL TABLES

Table S1: Demographic and clinical characteristics of the patient cohort.

Table S2. List of antibodies used for immunohistochemistry.

Table S3. List of biopsies used for immunohistochemistry, gene array analysis and digital special profiling.

Table S4. List of morphology markers used for digital spatial profiling.

Table S5. List of 48 psoriasis-defining genes.

Table S6. List of GeoMx Human Immuno-Oncology protein assays used in the study.

SUPPLEMENTAL FIGURES

Figure S1. Segmented immunohistochemistry results from Ki67, CD3, CD4, and CD8 from the two cohorts at baseline and end of treatment.

ab. Total Ki67$^+$ and dermal Ki67$^+$ cells. cd. Epidermal and dermal CD3$^+$ cells. ef. Epidermal and dermal CD4$^+$ cell quantity measurements. gh. Epidermal and dermal CD8$^+$ cell quantity measurements. Mean ± SD depicted. One-way analysis of variance (ANOVA) with Tukey's multiple comparison test was used to compare across cohorts and repeated measures analysis of variance with the post hoc Šidák test was used to compare between timepoints within studies. *P < 0.05, **P < 0.01, ***P < 0.001, ****P < 0.0001. End of treatment (EOT), nonlesional (NL), lesional (LS), Dead Sea climatotherapy (DSC), secukinumab (SEC).

Figure S2. Immunohistochemical results from CD1a, CD11c, CD45RO and myeloperoxidase from the two cohorts at baseline and end of treatment.

a. Results from CD1a$^+$ staining. b. Results from CD11c$^+$ staining. c. Results from CD45RO$^+$ staining. d. Results from myeloperoxidase (MPO). The dashed line indicates the interface between epidermis and dermis. Sequential slides from the same patient are shown. Scale bars = 200 µm. Mean ± SD depicted. One-way analysis of variance (ANOVA) with Tukey's multiple comparison test was used to compare across cohorts and repeated measures analysis of variance with the post hoc Šidák test was used to compare between timepoints within studies. *P < 0.05, **P < 0.01, ***P < 0.001, ****P < 0.0001. End of treatment (EOT), nonlesional (NL), lesional (LS), Dead Sea climatotherapy (DSC), secukinumab (SEC).

Figure S3. Immunohistochemical results from CD1a, CD11c, CD45RO, and myeloperoxidase from the two cohorts at baseline and end of treatment separated into an epidermal and dermal segment.

a-h. Results from quantitative immunohistochemistry analysis of $CD1a^+$, $CD11c^+$, $CD45RO^+$, and MPO^+ cells from nonlesional (NL) and lesional (LS) skin taken at baseline and end of treatment (EOT) from patients treated with Dead Sea climatotherapy (DSC) and patients treated with secukinumab (SEC). Mean ± SD depicted. One-way analysis of variance (ANOVA) with Tukey's multiple comparison test was used to compare across cohorts and repeated measures analysis of variance with the post hoc Šidák test was used to compare between timepoints within studies. For *P < 0.05, **P < 0.01, ***P < 0.001, ****P < 0.0001.

Figure S4. Immunohistochemical results from FOXP3, CD163, CD15, CD56, CD103, and CD207 from the two cohorts at baseline and end of treatment separated into an epidermal and dermal segment.

a-l. Results from quantitative immunohistochemistry analysis of $FOXP3^+$, $CD163^+$, $CD15^+$, $CD56^+$, $CD103^+$, and $CD207^+$ cells from nonlesional (NL) and lesional (LS) skin taken at baseline and end of treatment (EOT) from patients treated with Dead Sea climatotherapy (DSC) and patients treated with secukinumab (SEC). Mean ± SD depicted. One-way analysis of variance (ANOVA) with Tukey's multiple comparison test was used to compare across cohorts and repeated measures analysis of variance with the post hoc Šidák test was used to compare between timepoints within studies. For *P < 0.05, **P < 0.01, ***P < 0.001, ****P < 0.0001.

Figure S5. Clustering of all samples according to time and treatment.

a. Heatmap and two-way semi-supervised clustering based on 2,735 differentially expressed genes (Var>0.1, p<0.05, q=0.06, >2-FC, paired analysis) between baseline LS and (LS EOT & baseline NL). The samples are colored according to time (baseline LS, gray; baseline NL, brown; EOT-SEC, turquoise; EOT-DSC, purple) and study (DSC, black; SEC, cyan). The

colors in the heatmap signify high (yellow) or low (blue) expression of a particular gene across samples (z-scaled values). A clear normalization of the psoriasis baseline (left cluster) signature is observed with time and treatment (right cluster, where the LS EOT samples cluster together with the baseline NL samples). b. Sample PCA plot based on the same 2735 differentially expressed genes as for the heatmap. The 44 samples are colored according to (top) time (baseline LS, gray; baseline NL, brown; EOT-SEC, turquoise; EOT-DSC, purple) or (bottom) study (DSC, black; SEC, cyan).

Figure S6. The transcriptome of nonlesional (NL) skin versus lesional (LS) psoriasis skin in the two cohorts.

a. Heatmap and two-way semi-supervised clustering based on 2,751 differentially expressed genes (DEGs) (Var>0.1, p<0.05, q=0.07, >2-FC) between all baseline LS and NL samples. The samples are colored according to tissue (LS, gray; NL, brown) and study (DSC, black; SEC, cyan). The colors in the heatmap signify high (yellow) or low (blue) expression of a particular gene across samples (z-scaled values). Clear separation of baseline LS (left cluster) from NL (right cluster) samples is observed. b. Volcano plot showing the log2 (fold difference) between the baseline LS and NL group on the x-axis and the -log10 of the p-value for the two groups (baseline LS vs. NL) t-test on the y-axis. 2,751 genes meet the cut-off criteria, (p<0.05, >2-FC), selected DEGs are indicated. c-d. Venn diagram showing the overlap between the pooled (SEC-DSC) and individual baseline LS/NL contrasts for DEG-up (c) and DEG-down (d).

Figure S7. Top canonical pathways, diseases and bio functions based on all differentially expressed genes Found in the baseline nonlesional vs. baseline lesional contrast.

a. Top canonical pathways based on the differentially expressed genes (DEGs) observed in the baseline nonlesional (NL) vs. baseline lesional (LS) comparison. b. Top diseases and bio functions based on the differentially expressed genes observed in the baseline NL vs. baseline LS comparison.

Figure S8. The transcriptome of psoriasis in baseline nonlesional skin versus baseline lesional skin in patients treated with Dead Sea climatotherapy and secukinumab.

a. Heatmap and two-way semi-supervised clustering based on 2,308 differentially expressed genes (DEGs) (Var>0.1, p<0.05, q=0.07, >2-FC) between Dead Sea climatotherapy (DSC) baseline LS and NL samples. The samples are colored according to tissue (LS, gray; NL, brown). The colors in the heatmap signify high (yellow) or low (blue) expression of a particular gene across samples (z-scaled values). Clear separation of baseline LS (left cluster) from NL (right cluster) samples is observed. b. Volcano plot showing the log2(fold difference) between the DSC baseline LS and NL groups on the x-axis and the -log10 of the p-value for the two group (baseline LS vs. NL) paired t-test on the y-axis. 2,308 genes meet the cut-off criteria, (p<0.05, >2-FC), selected DEGs are indicated. c. Heatmap and two-way semi-supervised clustering based on 2,496 DEGs (Var>0.1, p<0.05, q=0.08, >2-FC) between SEC baseline LS and NL samples. The samples are colored according to tissue (LS, gray; NL, brown). The colors in the heatmap signify high (yellow) or low (blue) expression of a particular gene across samples (z-scaled values). Clear separation of baseline LS (left cluster) from NL (right cluster) samples is observed, except for Pt12. d. Volcano plot showing the log2 (fold difference) between the SEC baseline LS and NL group on the x-axis and the -log10 of the p-value for the two group (baseline LS vs. NL) paired t-test on the y-axis. 2496 genes meet the cut-off criteria, (p<0.05, >2-FC), selected DEGs are indicated.

Figure S9. The transcriptome of psoriasis in baseline nonlesional versus end of treatment lesional skin in patients treated with Dead Sea climatotherapy and secukinumab.

a. Heatmap and two-way semi-supervised clustering based on 180 DEGs (Var>0.1, q<0.20, p<0.02, >2-FC) between DSC LS-EOT and NL-D0 samples. The samples are colored according to tissue (LS, gray; NL, brown). The colors in the heatmap signify high (yellow) or low (blue) expression of a particular gene across samples (z-scaled values). b. Volcano plot showing the log2(fold difference) between the DSC LS-EOT and NL-D0 group on the x-axis and the -log10 of the p-value for the two groups (LS-EOT vs. NL-D0) paired t-test on the y-axis. 180 genes meet the cut-off criteria, (q<0.20, >2-FC), selected DEG are indicated. c. Heatmap and two-way semi-supervised clustering based on 377 DEG (Var>0.1, q<0.20, p<0.055, >2-FC) between SEC LS-EOT and NL-D0 samples. The samples are colored according to tissue (LS, gray; NL, brown). The colors in the heatmap signify high (yellow) or low (blue) expression of a particular gene across samples (z-scaled values). d. Volcano plot showing the log2(fold difference) between the SEC LS-EOT and NL-D0 group on the x-axis and the -log10 of the p-value for the two groups (LS-EOT vs. NL-D0) paired t-test on the y-axis. 377 genes meet the cut-off criteria, (q<0.20, >2-FC), selected DEG are indicated.

Figure S10. Pathway analysis performed on the differentially expressed genes in baseline nonlesional skin versus end of treatment lesional skin in patients treated with Dead Sea climatotherapy.

a. Top canonical pathways based on the differentially expressed genes (DEGs) observed in the baseline nonlesional (NL) vs end of treatment lesional (EOT LS) comparison. b. The IL-17A signaling pathway in fibroblasts and overlapping signature with other pathways. c. Top

diseases and bio functions based on the differentially expressed genes observed in the baseline NL vs EOT LS comparison.

Figure S11. Pathway analysis performed on the differentially expressed genes in baseline nonlesional skin versus end of treatment lesional skin in patients treated with secukinumab.

a. Top canonical pathways based on the differentially expressed genes (DEGs) observed in the baseline nonlesional (NL) vs. baseline end of treatment lesional (EOT LS) comparison. b. Top diseases and bio functions based on the differentially expressed genes observed in the baseline NL vs. EOT LS comparison.

Figure S12. Digital spatial profiling of complete responders from the two cohorts.

a. Image showing the setup on the slides with baseline nonlesional (NL), baseline lesional (LS), and end of treatment (EOT) LS. Size bar = 2 mm. b. Larger image of a baseline LS region of interest showing the epidermis area of illumination (AOI) in red overlay and the $CD103^+$ AOI in the green overlay using fluorescence-based segmentation. c. Larger image of a ROI showing an example of the $CD103^+$ cell microenvironment AOI. d. Example of an AOI including a dermal infiltrate. e. AOI showing the top of an epidermal papillae. The white dashed lines indicate the interface between the epidermis and dermis. Size bars = 100 µm.

Figure S13. Heatmap and unsupervised hierarchical clustering of all the AOIs and proteins used for the digital spatial profiling experiment.

All samples used for the digital spatial profiler experiment. The relative expression is delineated by a scale from yellow (activation) to blue (inhibition).

Figure S14. Differentially expressed proteins at baseline nonlesional, baseline lesional, and end of treatment lesional skin between the two cohorts.

a. Differentially expressed proteins (DEPs) in the $CD103^+$ microenvironment in secukinumab (SEC) treated patients between baseline nonlesional (NL) and end of treatment (EOT) lesional (LS) skin. b. DEPs in the dermal infiltrate in SEC treated patients between baseline NL and EOT LS skin. c. DEPs in the epidermis in SEC treated patients between baseline NL and EOT LS skin. d. DEPs in the $CD103^+$ microenvironment in Dead Sea climatotherapy (DSC) treated patients between baseline NL and EOT LS skin. e. DEPs in the dermal infiltrate in DSC treated patients between baseline NL and EOT LS skin. f. DEPs in the epidermis in DSC treated patients between baseline NL and EOT LS skin. g. DEPs in the $CD103^+$ microenvironment in SEC treated patients between baseline NL and baseline LS skin. h. DEPs in the dermal cell infiltrate in SEC treated patients between baseline NL and baseline LS skin. i. DEPs in the epidermis in SEC treated patients between baseline NL and baseline LS skin.

References:

1. Bankhead, P.; Loughrey, M.B.; Fernández, J.A.; Dombrowski, Y.; McArt, D.G.; Dunne, P.D.; McQuaid, S.; Gray, R.T.; Murray, L.J.; Coleman, H.G.; et al. QuPath: Open Source Software for Digital Pathology Image Analysis. *Sci Rep* **2017**, *7*, 16878, doi:10.1038/s41598-017-17204-5.
2. Emmanuel, T.; Brent, M.B.; Iversen, L.; Johansen, C. Quantification of Immunohistochemically Stained Cells in Skin Biopsies. *Dermatopathology (Basel)* **2022**, *9*, 82–93, doi:10.3390/DERMATOPATHOLOGY9020011.
3. Ignatov, B.; Sortebech, D.; Emmanuel, T.; Zhuravleva, E.; Eidsmo, L. Method for High-plex Analysis of Immune Cells in Human Skin Using the GeoMx System. *Scand J Immunol* **2023**, doi:10.1111/sji.13326.
