# Supplementary material for: Secukinumab and Dead Sea Climatotherapy Impact Resolved Psoriasis Skin Differently Potentially Affecting Disease Memory
Source: Int J Mol Sci. 2024 May 31;25(11):6086. doi: 10.3390/ijms25116086 (PMC11172747; doi:10.3390/ijms25116086)
Supplement: Supplementary file 1 [file ijms-25-06086-s001.zip › table_s1.pdf]

	Dead Sea climatotherapy							Mean ± SD or median, [IQR]	Secukinumab								Mean ± SD or median, [IQR]	p-value
Patient no.	1	4	8	9	13	14	19	-	2	5	8	9	11	12	13	14	-	-
Age, *yr*	57	38	52	45	57	50	62	51.6 ± 8.1	53	26	36	38	34	48	45	40	40.0 ± 8.5	**0.0191**†
Gender	F	M	M	M	F	M	M	-	M	F	M	F	M	M	M	F	-	>0.9999*
BMI, *kg/m^2*	34.2	-	29.6	25.7	34.1	46.1	27.7	32.9 ± 7.3	-	44.1	24.6	24.4	26.5	31.3	38.4	30.8	31.4 ± 7.4	0.7289†
Smoker	-	Yes	-	No	-	-	-	-	Ex	No	No	No	Yes	Ex	Ex	No	-	-
Comorbidities	Anxiety, depression, DM, hypertension, osteoarthritis	Hypercholesterolemia, hypertension, osteoarthritis	None	Anxiety, DM Hypercholesterolemia, hypertension	DM, Hypertension	DM, hypertension	None	-	DM, hypercholesterolemia	PCO	None	None	None	PTSD, hypertension, NASH	Hypertension	None	-	-
Psoriasis arthritis	Yes	Yes	Yes	No	Yes	No	Yes	-	No	No	Yes	Yes	Yes	Yes	Yes	Yes	-	>0.9999*
Age of psoriasis onset, *yr*	7	15	24	28	12	11	6	14.7 ± 8.4	29	13	19	18	32	-	-	15	21.0 ± 7.7	0.1894†
Baseline PASI	11.5	10.6	11.6	23.6	17	18.7	11.4	11.6, [11.4-18.7]	19.2	18.3	28	23.1	17.6	51.8	13.8	15.6	18.8, [16.1-26.8]	0.0721‡
Medication ≤4 weeks prior to or at baseline	None	Topical steroids	Topical steroids	None	Topical steroids	None	None	-	None	None	None	None	None	None	None	None	-	-
Prior treatments	Topical steroids, UVB, tar, mtx, Enbrel, Humira, Remicade, climatotherapy	Topical steroids, UVB, mtx, climathotherapy	Topical steroids, mtx, climathotherapy	Topical steroids, UVB, mtx, climathotherapy	Topical steroids, UVB, climathotherapy	Topical steroids, UVB, PUVA, tar, mtx, climatotherapy	Topical steroids, carbon-arc light, tar, mtx, Humira, Cimzia,	-	Topical steroids, UVB, Bucky, tar, mtx	Topical steroids, UVB, mtx	Topical steroids, mtx, climatotherapy,	Topical steroids, Bucky, mtx, neotigason	Topical steroids, mtx	Topical steroids, mtx, imurel, ciclosporin	Topical steroids, UVB, mtx, neotigason	Topical steroids, UVB, mtx	-	-
C-reactive protein, *mg/L*	4.1	1.6	6.5	1.7	1.4	6.3	23.5	4.1, [1.6-6.5]	5	16	7.4	8.4	2.9	4	2.5	2	4.5, [2.6-8.2]	0.5358‡
Cholesterol, *mmol/L*	4.0	7.1	5.7	5.7	6.0	4.1	4.5	5.3 ± 1.1	-	5.1	4.2	4.2	6.8	6.0	4.6	4.2	5.0 ± 1.0	0.6314†
Triglyceride, *mmol/L*	3.4	2.7	1.3	2.6	3.2	4.1	0.7	2.6 ± 1.2	-	1.6	0.8	0.9	2.3	3.2	1.4	-	1.7 ± 0.9	0.1731†
HbA1c > 48, *mmol/mol*	Yes	No	No	Yes	Yes	Yes	Yes	-	-	No	No	No	No	No	No	No	-	**0.0210***
Glucose, *mmol/L*	4.8	5.3	5.7	20.7	6.3	12.7	8.1	6.3, [5.3-12.7]	-	5.5	5.6	4.7	5.2	7.2	4.8	6.1	5.5, [4.8-6.1]	0.1352‡

Abbreviations: DM = Diabetes mellitus, NASH = Nonalcoholic steatohepatitis, MTX = Methotrexate, PCO = Polycystic ovary disease, PTSD = Post-traumatic stress disorder. *Chi-squared test. † Students t-test. ‡ Mann-Whitney *U* test. § CRP measurements below 0.6 were set to 0.6. Underlined and **bold** indicates statistical significance.
