# Supplementary material for: Secukinumab and Dead Sea Climatotherapy Impact Resolved Psoriasis Skin Differently Potentially Affecting Disease Memory
Source: Int J Mol Sci. 2024 May 31;25(11):6086. doi: 10.3390/ijms25116086 (PMC11172747; doi:10.3390/ijms25116086)
Supplement: Supplementary file 1 [file ijms-25-06086-s001.zip › table_s2.pdf]

Marker	Cell	Clone	Lot	Dilution	Incubation length	Vendor	Isotype	Cat Number	RRID
CD1a	Langerhans cell	O10	1856P2012F	1:150	30 min	Lab Vision/ Epredia	Mouse Monoclonal $IgG_{1/\kappa}$	MS-1856	Lab Vision Cat# MS-1856-P, RRID:AB_149548
CD3	T cell	MRQ-39	44612	1:200	30 min	Cell Marque	Rabbit Monoclonal, IgG_1	103R-96	Nolan lab – Stanford Cat# 103R, RRID:AB_2864399
CD4	Helper T cell	SP35	71055	1:250	30 min	Cell Marque	Rabbit Monoclonal, IgG	104R-16	Cell Marque, Cat# 104R, RRID:AB_1516770
CD8	Cytotoxic T cell	SP16	51708	1:500	30 min/ overnight	Cell Marque	Rabbit Monoclonal, IgG_1	108R-16	Cell Marque Cat# 108R, RRID:AB_2892088
CD11c	Dendritic cell	EP157	61438	1:50	30 min	Cell Marque	Rabbit Monoclonal, IgG	111R-16	Cell Marque Cat# 111R, RRID:AB_2892656
CD15	Myeloid cell	MMA	45963	1:100	30 min	Cell Marque	Mouse Monoclonal, IgM	115M-14	Cell Marque Cat# 115M-14, RRID:AB_1157919
CD45RO	Memory T cell	UCHL-1	129773	1:100	30 min	Cell Marque	Mouse Monoclonal, $IgG_{2a/\kappa}$	147M-94	Cell Marque, Cat# 147M, RRID:AB_1158175
CD49a	Skin resident cell	CL7207	MAB-03667	1:500	Overnight	Novus	Mouse Monoclonal, IgG	NBP2-76478	Novus Cat# NBP2-76478, RRID:AB_2893115
CD56	Natural killer cell	MRQ-42	106869	1:100	30 min	Cell Marque	Rabbit Monoclonal, IgG_1	156R-96	Cell Marque, Cat# 156R, RRID:AB_2861293
CD103	Skin resident cell	EP206	105295	1:60	30 min	Cell Marque	Rabbit Monoclonal, IgG	437R-15	Cell Marque Cat# 437R, RRID:AB_2884943
CD163	Macrophage	MRQ-26	93644	1:75	30 min	Cell Marque	Mouse Monoclonal, IgG_1	163M-15	Cell Marque Cat# 163M-15, RRID:AB_1159114
CD207	Langerhans cell	12D6	70437	1:100	30 min	Cell Marque	Mouse Monoclonal, $IgG_{2b/\kappa}$	392M-15	Millipore Cat# 392M-1, RRID:AB_2889342
FOXP3	Regulatory cell	EP340	20082419	1:100	30 min	Cell Marque	Rabbit Monoclonal, IgG	AC-0304RUOC	Cell Marque Cat# AC-0304RUO, RRID:AB_2892654
Ki67	Proliferation	SP6	62103	1:200	20 min	Cell Marque	Rabbit Monoclonal, IgG	275R-16	Cell Marque Cat# 275R-16, RRID:AB_1158037
MPO	Neutrophil	SP72	46344	1:225	Overnight	Cell Marque	Rabbit Monoclonal, IgG_1	289R-14	Cell Marque Cat# 289R, RRID:AB_2892655

Abbreviations: CD, cluster of differentiation; FOXP3, forkhead box P3; MPO, myeloperoxidase; RRID, research resource identifier.
