# Supplementary material for: Secukinumab and Dead Sea Climatotherapy Impact Resolved Psoriasis Skin Differently Potentially Affecting Disease Memory
Source: Int J Mol Sci. 2024 May 31;25(11):6086. doi: 10.3390/ijms25116086 (PMC11172747; doi:10.3390/ijms25116086)
Supplement: Supplementary file 1 [file ijms-25-06086-s001.zip › table_s3.pdf]

	Dead Sea climatotherapy							Secukinumab							
Patient no.	1	4	8	9	13	14	19	2	5	8	9	11	12	13	14
Biopsy site region	Left gluteal	Left hip	Right lower back	Left upper arm	Right gluteal	Left upper arm	Left gluteal	Left anterior thigh	Right leg	Left gluteal	Right gluteal	Right elbow	Right gluteal	Left gluteal	Right lower back
IHC	X	X	X	X	X	X	X	X	(X)	X	X	X		X	X
Gene array	X	X	X	X	X	X	X	X	X	X	X	X	(X)	X	X
DSP Exp 1		X		X						X				X	
DSP Exp 2	X	X	X	X	X	X	X	X	X	X	X	X		X	X

Abbreviations: DSP, digital spatial profiling; IHC, immunohistochemistry.
