# Supplementary material for: Secukinumab and Dead Sea Climatotherapy Impact Resolved Psoriasis Skin Differently Potentially Affecting Disease Memory
Source: Int J Mol Sci. 2024 May 31;25(11):6086. doi: 10.3390/ijms25116086 (PMC11172747; doi:10.3390/ijms25116086)
Supplement: Supplementary file 1 [file ijms-25-06086-s001.zip › table_s4.pdf]

Marker	Cell	Clone	Lot	Dilution	Incubation length	Vendor	Isotype	Cat Number	RRID	Flourophore
CD3	T cell	CD3-12	-	1:200	Overnight	Bio-Rad	Rat Monoclonal, IgG_1	MCA1477A647	Bio-Rad Cat# MCA1477A647, RRID:AB_10841760	Alexa Fluor® 647
CD8A	Helper T cell	OTI3H6	-	1:100	Overnight	OriGene	Mouse Monoclonal, IgG_1	CF802079	OriGene, Cat# CF802079, RRID:AB_2923423	Self conjugated, Alexa Fluor® 647
CD45	Leucocytes	D9M8I	-	1:40	Overnight	Cell Signaling Technology	Rabbit Monoclonal, IgG	13917	Cell Signaling Technology Cat# 13917, RRID:AB_2750898	Alexa Fluor® 594
CD103	Skin resident cell	ITGAE/3904R	-	1:100	Overnight	Novus Biologicals	Rabbit Monoclonal, IgG	NBP3-08855DL594	Novus, Cat# NBP3-08855DL594, RRID:AB_2923422	DyLight™ 594
MelanA	Melanocytes	A103	-	1:100	Overnight	Santa Cruz Biotechnology	Mouse Monoclonal $IgG_{1/\kappa}$	sc-20032	Santa Cruz Biotechnology Cat# sc-20032, RRID:AB_627912	Texas Red 615
Syto 13	Nucleic Acid Stain	-	-	200nM	Overnight	Invitrogen	-	S7575	-	SYTO™13
Syto 83	Nucleic Acid Stain	-	-	200nM	Overnight	Invitrogen	-	S11364	-	SYTO™83

Abbreviations: CD, cluster of differentiation; RRID, research resource identifier.
