# Supplementary material for: Secukinumab and Dead Sea Climatotherapy Impact Resolved Psoriasis Skin Differently Potentially Affecting Disease Memory
Source: Int J Mol Sci. 2024 May 31;25(11):6086. doi: 10.3390/ijms25116086 (PMC11172747; doi:10.3390/ijms25116086)
Supplement: Supplementary file 1 [file ijms-25-06086-s001.zip › table_s6.pdf]

Immune Cell Profiling Panel				
**Protein Name**	**Gene**	**Accession**	**Full Target Name**	**Protein ID**
PD-1	PDCD1	A0A0M3M0G7, Q15116	programmed cell death 1	DPROT_00004.1
CD68	CD68	P34810	CD68 molecule	DPROT_00006.1
HLA-DR	CD74	P04233	CD74 molecule, major histocompatibility complex, class II invariant chain	DPROT_00007.1
Ki-67	MKI67	P46013	marker of proliferation Ki-67	DPROT_00009.1
Beta-2-microglobulin	B2M	P61769	beta-2-microglobulin	DPROT_00010.1
CD11c	ITGAX	P20702, H3BN02	integrin, alpha X (complement component 3 receptor 4 subunit)	DPROT_00011.1
CD20	MS4A1	A0A024R507, P11836	membrane-spanning 4-domains, subfamily A, member 1	DPROT_00012.1
CD3	CD3G, CD3E, CD3D	B0YIY5, P09693, P07766, B0YIY4, P04234	CD3g molecule, gamma (CD3-TCR complex), CD3e molecule, epsilon (CD3-TCR complex) CD3d molecule, delta (CD3-TCR complex)	DPROT_00013.1
CD4	CD4	B4DT49, P01730, B0AZV7	CD4 molecule	DPROT_00014.1
CD45	PTPRC	M9MML4, A0A0A0MT22, P08575, X6R433	protein tyrosine phosphatase, receptor type, C	DPROT_00015.1
CD56	NCAM1	P13591, A0A087WWD4	neural cell adhesion molecule 1	DPROT_00016.1
CD8	CD8A	P01732, Q6ZVS2, Q8TAW8	CD8a molecule	DPROT_00017.1
CTLA4	CTLA4	P16410	cytotoxic T-lymphocyte associated protein 4	DPROT_00395.1
GZMB	GZMB	J3KQ52, Q6XGZ4, P10144, Q67BC3	granzyme B	DPROT_00019.1
PD-L1	CD274	Q0GN75, Q9NZQ7	CD274 molecule	DPROT_00021.1
PanCk	KRT1, KRT10, KRT2, KRT16, KRT5, KRT6B,	P04264, P13645, P35908, P08779, P13647, P04259, P08727, A0A0S2Z428, P02538, Q7L4M3, P05787, P02533, P12035	Keratin 1, keratin 10, keratin 2, keratin 16, keratin 5, keratin 6B, keratin 19, keratin 6A, keratin 8, keratin 14, keratin 3.	DPROT_00022.1

	KRT19,<br>KRT6A,<br>KRT8,<br>KRT14,<br>KRT3			
SMA	ACTA2	D2JYH4, P62736	actin, alpha 2, smooth muscle, aorta.	DPROT_00023.1
Fibronectin	FN1	B7ZLE5, P02751,<br>Q6MZM7,<br>Q9UQS6, Q6MZF4,<br>Q6N084	fibronectin 1	DPROT_00024.1
Ms IgG1	-	-	-	DPROT_00002.1
Ms IgG2a	-	-	-	DPROT_00003.1
Rb IgG	-	-	-	DPROT_00001.1
GAPDH	GAPDH	P04406, V9HVZ4	glyceraldehyde-3-phosphate dehydrogenase	DPROT_00020.1
Histone H3	H3C1	P68431	H3 clustered histone 1	DPROT_00005.1
S6	RPS6	P62753, A2A3R6	ribosomal protein S6	DPROT_00008.1
**IO Drug Target Panel**				
**Protein Name**	**Gene**	**Accession**	**Full Target Name**	**Protein ID**
4-1BB	TNFRSF9	Q07011	tumor necrosis factor receptor superfamily, member 9	DPROT_00026.1
LAG3	LAG3	P18627	lymphocyte-activation gene 3	DPROT_00027.1
OX40L	TNFSF4	P23510, A0A024R937	tumor necrosis factor superfamily member 4	DPROT_00028.1
Tim-3	HAVCR2	Q8TDQ0	hepatitis A virus cellular receptor 2	DPROT_00029.1
VISTA	VSIR	Q9H7M9	V-set immunoregulatory receptor	DPROT_00030.1
ARG1	ARG1	P05089	arginase 1	DPROT_00031.1
B7-H3	CD276	Q5ZPR3, A0A0C4DGH0,<br>A8K9J6	CD276 molecule	DPROT_00032.1
IDO1	IDO1	P14902	indoleamine 2,3-dioxygenase 1	DPROT_00033.1
STING	TMEM173	V5V0K2, Q86WV6	transmembrane protein 173	DPROT_00034.1
GITR	TNFRSF18	Q9Y5U5, A0A0R7FDM1	TNF receptor superfamily member 18	DPROT_00035.1
**Immune Activation Status Panel**				

Protein Name	Gene	Accession	Full Target Name	Protein ID
CD127	IL7R	P16871	interleukin 7 receptor	DPROT_00036.1
CD25	IL2RA	P01589, Q5W005	interleukin 2 receptor, alpha	DPROT_00037.1
CD80	CD80	A0N0P2, P33681	CD80 molecule	DPROT_00038.1
ICOS	ICOS	Q53QY6, Q9Y6W8	inducible T-cell co-stimulator	DPROT_00040.1
PD-L2	PDCD1LG2	Q9BQ51	programmed cell death 1 ligand 2	DPROT_00041.1
CD40	CD40	A0A0S2Z3C7, P25942, Q6P2H9, A0A0S2Z349	CD40 molecule, TNF receptor superfamily member 5	DPROT_00042.1
CD44	CD44	P16070	CD44 molecule (Indian blood group)	DPROT_00044.1
CD27	CD27	P26842	CD27 molecule	DPROT_00045.1
**Pan-Tumor Panel**				
**Protein Name**	**Gene**	**Accession**	**Full Target Name**	**Protein ID**
MART1	MLANA	Q16655	melan-A	DPROT_00054.1
NY-ESO-1	CTAG1B	P78358	cancer/testis antigen 1B	DPROT_00055.1
S100B	S100B	A0A0S2Z4C5, P04271	S100 calcium binding protein B	DPROT_00056.1
Bcl-2	BCL2	A0A024R2B3, P10415, A0A024R2C4	B-cell CLL/lymphoma 2	DPROT_00057.1
EpCAM	EPCAM	P16422	epithelial cell adhesion molecule	DPROT_00058.1
Her2	ERBB2	P04626, J3QLU9, F5H1T4, X5DNK3	v-erb-b2 avian erythroblastic leukemia viral oncogene homolog 2	DPROT_00059.1
PTEN	PTEN	F6KD01, P60484	phosphatase and tensin homolog	DPROT_00060.1
ER-alpha	ESR1	G4XH65, P03372, Q9UBT1, A8KAF4, H0Y4W6, A0A125SXW3	estrogen receptor 1	DPROT_00061.1
PR	PGR	P06401	progesterone receptor	DPROT_00062.1
**PI3K/AKT Signaling Panel**				
**Protein Name**	**Gene**	**Accession**	**Full Target Name**	**Protein ID**
Phospho-AKT1 (S473)	AKT1	B0LPE5, P31749, B3KVH4	AKT serine/threonine kinase 1	DPROT_00236.1
Phospho-GSK3B (S9)	GSK3B	P49841, Q6FI27	glycogen synthase kinase 3 beta	DPROT_00238.1

Phospho-GSK3A (S21)/Phospho-GSK3B (S9)	GSK3A, GSK3B	P49841, Q6FI27, A0A024R0L5, P49840	glycogen synthase kinase 3 beta, glycogen synthase kinase 3 alpha	DPROT_00334.1
INPP4B	INPP4B	O15327, E7EQN9	inositol polyphosphate-4-phosphatase type II B	DPROT_00240.1
PLCG1	PLCG1	P19174, Q4LE43, Q9UFY1	phospholipase C gamma 1	DPROT_00241.1
Phospho-PRAS40 (T246)	AKT1S1	A0A024QZF6, Q96B36	AKT1 substrate 1	DPROT_00242.1
Phospho-Tuberin (T1462)	TSC2	P49815, X5D7Q2, B3KWH7, Q5HYF7, H3BMQ0	TSC complex subunit 2	DPROT_00243.1
Pan-AKT	AKT1, AKT2, AKT3	B0LPE5, P31749, B3KVH4, B4DG79, P31751, Q9Y243	AKT serine/threonine kinase 1, AKT serine/threonine kinase 2, AKT serine/threonine kinase 3	DPROT_00318.1
MET	MET	A0A024R759, P08581, A0A024R728, E6Y365, B4DLF5	MET proto-oncogene, receptor tyrosine kinase	DPROT_00332.1
Phospho-AKT (T308)	AKT1, AKT2	B0LPE5, P31749, P31751, B4DG79, B3KVH4	AKT serine/threonine kinase 1, AKT serine/threonine kinase 2	DPROT_00333.1
**MAPK Signaling Panel**				
**Protein Name**	**Gene**	**Accession**	**Full Target Name**	**Protein ID**
BRAF	BRAF	P15056	B-Raf proto-oncogene, serine/threonine kinase	DPROT_00226.1
EGFR	EGFR	Q504U8, E7BSV0, F2YGG7, E9PFD7, B7Z2I3, P00533	epidermal growth factor receptor	DPROT_00132.1
Phospho-c-RAF (S338)	RAF1	L7RRS6, P04049, A0A0S2Z4L5	v-raf-1 murine leukemia viral oncogene homolog 1	DPROT_00330.1
Phospho-JNK (T183/Y185)	MAPK8	A1L4K2, P45983	mitogen-activated protein kinase 8	DPROT_00229.1
Phospho-MEK1 (S217/S221)	MAP2K1	A4QPA9, Q02750, B4DFY5, H3BRW9	mitogen-activated protein kinase kinase 1	DPROT_00230.1
Phospho-p38 MAPK (T180/Y182)	MAPK14	L7RSM2, Q16539, A0A024RD15, B4E0K5	mitogen-activated protein kinase 14	DPROT_00231.1

Phospho-p44/42 MAPK ERK1/2 (T202/Y204)	MAPK3, MAPK1	P27361, Q9BWJ1, L7RXH5, P28482, Q1HBJ4, Q499G7	mitogen-activated protein kinase 3, mitogen-activated protein kinase 1	DPROT_00232.1
pan-RAS	HRAS, KRAS, NRAS	P01112, X5D945, A0A024RAV5, P01116, I1SRC5, L7RSL8, P01111, Q5U091	Harvey rat sarcoma viral oncogene homolog, KRAS proto-oncogene, GTPase, NRAS proto-oncogene, GTPase	DPROT_00222.1
p44/42 MAPK ERK1/2	MAPK3, MAPK1	P27361, Q9BWJ1, L7RXH5, P28482, Q1HBJ4, Q499G7	mitogen-activated protein kinase 3, mitogen-activated protein kinase 1	DPROT_00234.1
Phospho-p90 RSK (T359/S363)	RPS6KA1	Q15418	ribosomal protein S6 kinase A1	DPROT_00235.1
**Cell Death Panel**				
**Protein Name**	**Gene**	**Accession**	**Full Target Name**	**Protein ID**
BAD	BAD	A0A024R562, Q92934	BCL2-associated agonist of cell death	DPROT_00246.1
BCL6	BCL6	P41182, B5B0A5	B-cell CLL/lymphoma 6	DPROT_00247.1
BCLXL	BCL2L1	Q07817, Q5TE63, A0A0S2Z3C5	BCL2-like 1	DPROT_00248.1
CD95/Fas	FAS	P25445, K9J972, Q59FU8	Fas cell surface death receptor	DPROT_00249.1
GZMA	GZMA	P12544	granzyme A	DPROT_00215.1
Cleaved Caspase 9	CASP9	P55211, F8VVS7	caspase 9, apoptosis-related cysteine peptidase	DPROT_00337.1
p53	TP53	K7PPA8, P04637, Q53GA5, H2EHT1, A0A087X1Q1, A0A087WXZ1, A0A087WT22	tumor protein p53	DPROT_00129.1
PARP	PARP1	A0A024R3T8, P09874	poly	DPROT_00253.1
BIM	BCL2L11	O43521, A0A0A0MRE7, A0A0C4DH20	BCL2-like 11	DPROT_00254.1
