# Supplementary figures and images for: Secukinumab and Dead Sea Climatotherapy Impact Resolved Psoriasis Skin Differently Potentially Affecting Disease Memory
Source: Int J Mol Sci. 2024 May 31;25(11):6086. doi: 10.3390/ijms25116086 (PMC11172747; doi:10.3390/ijms25116086)

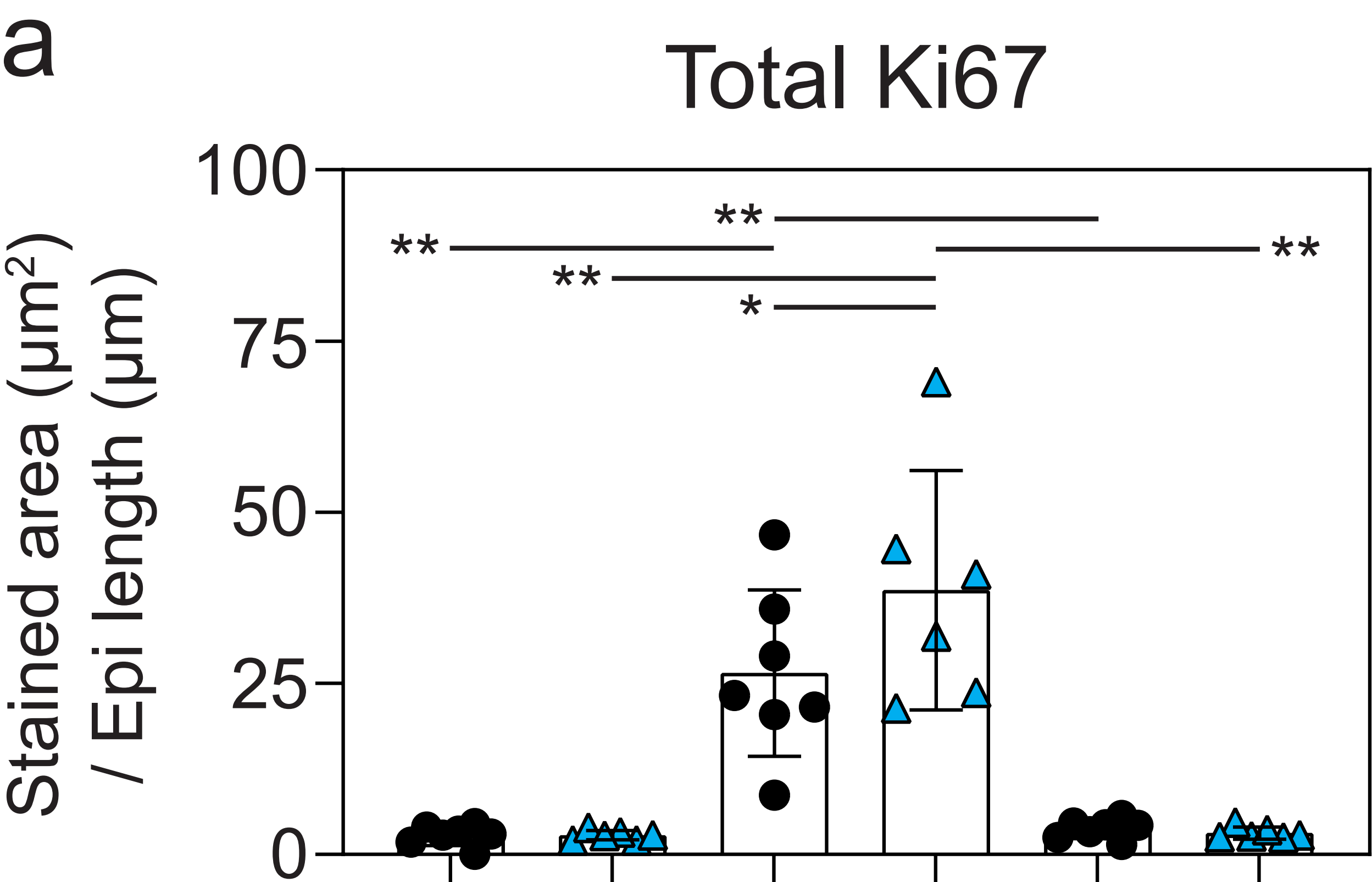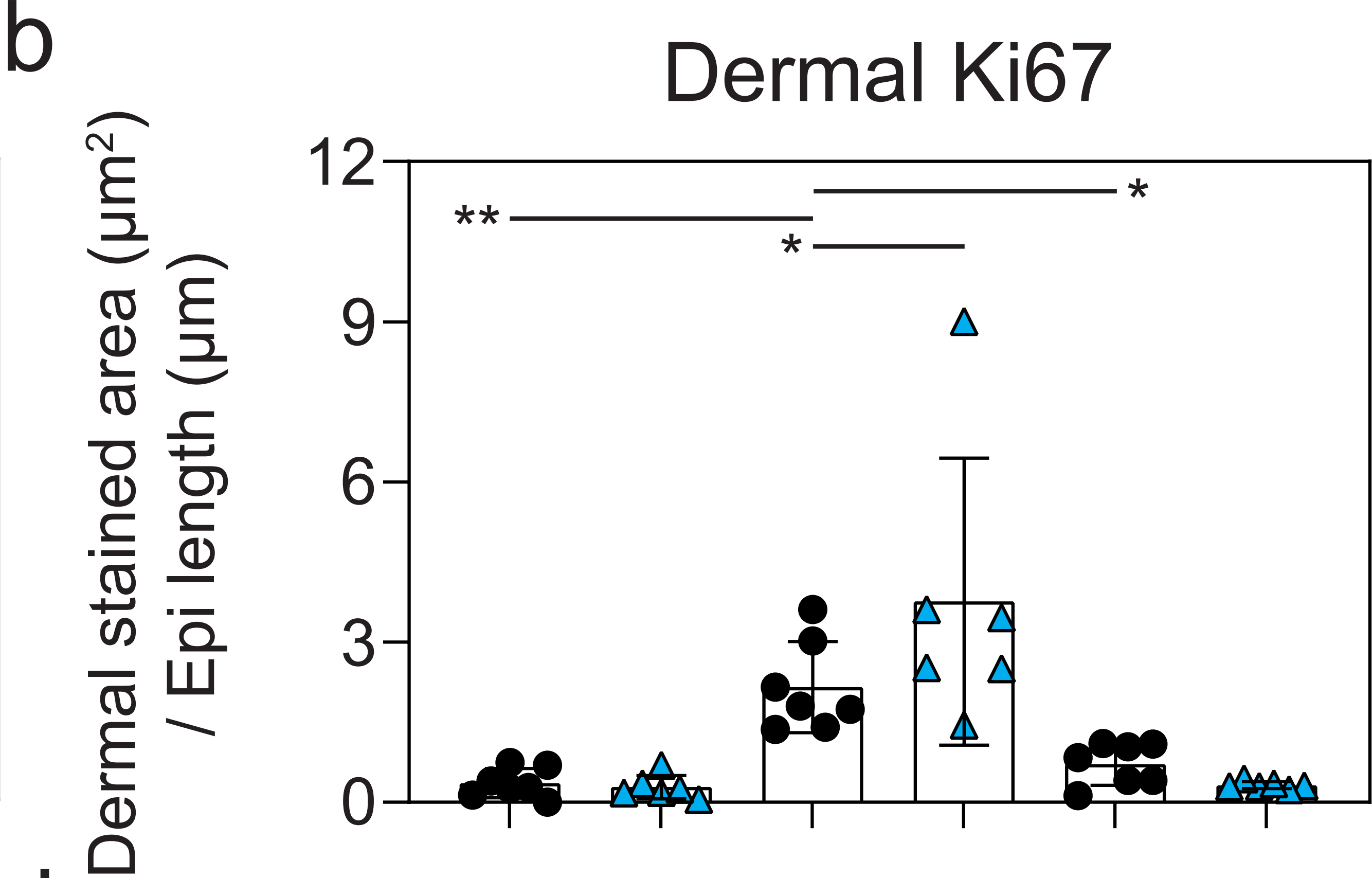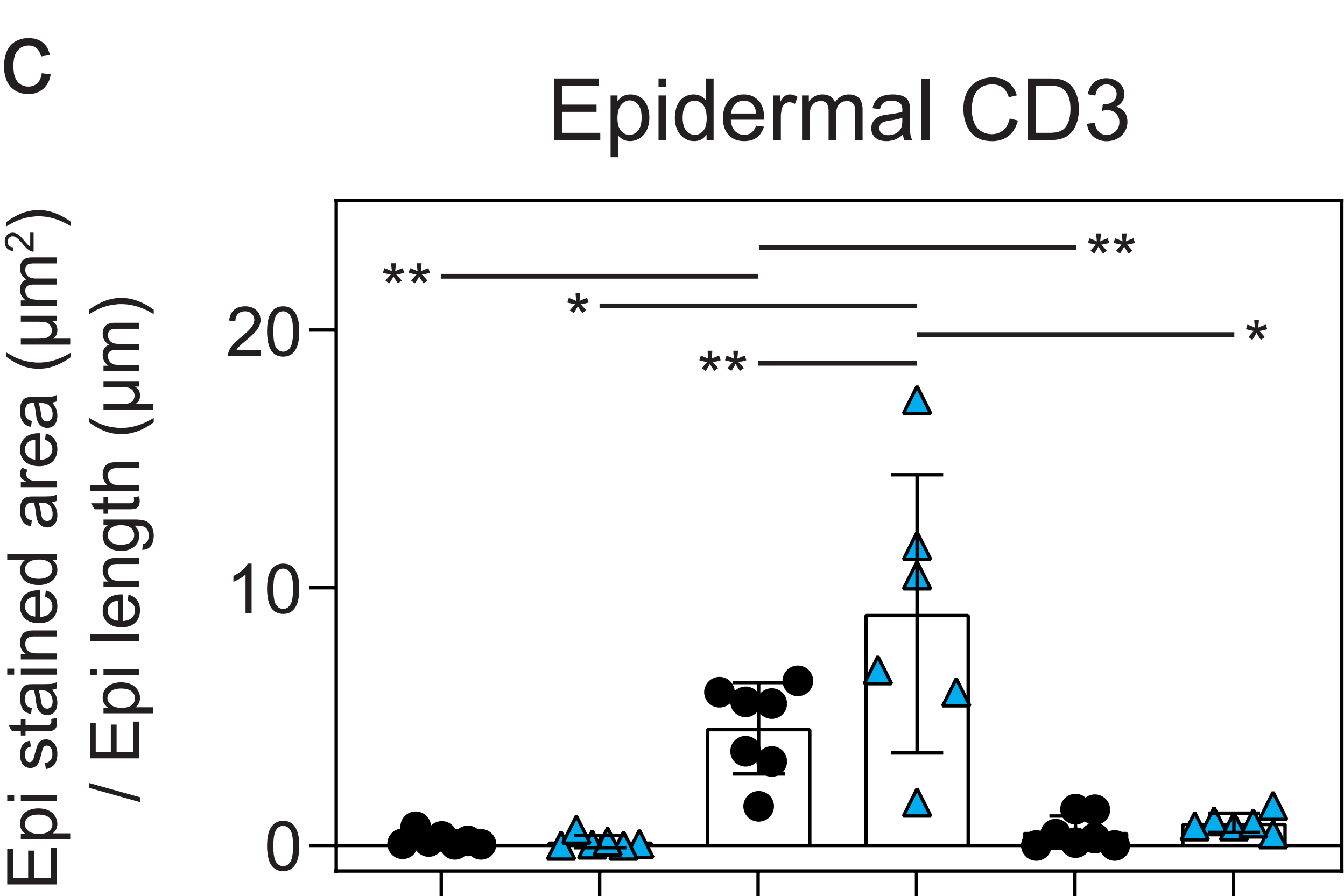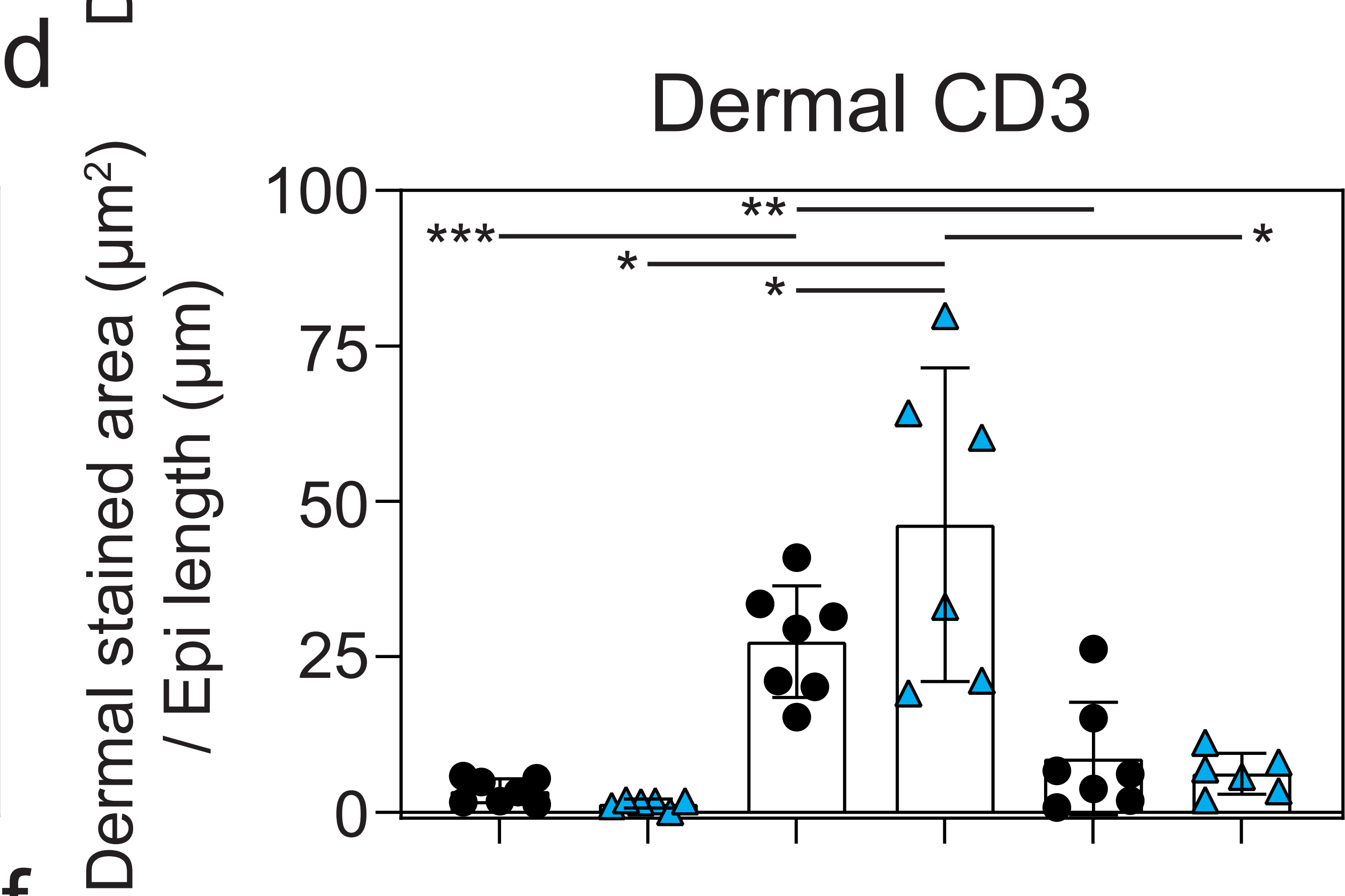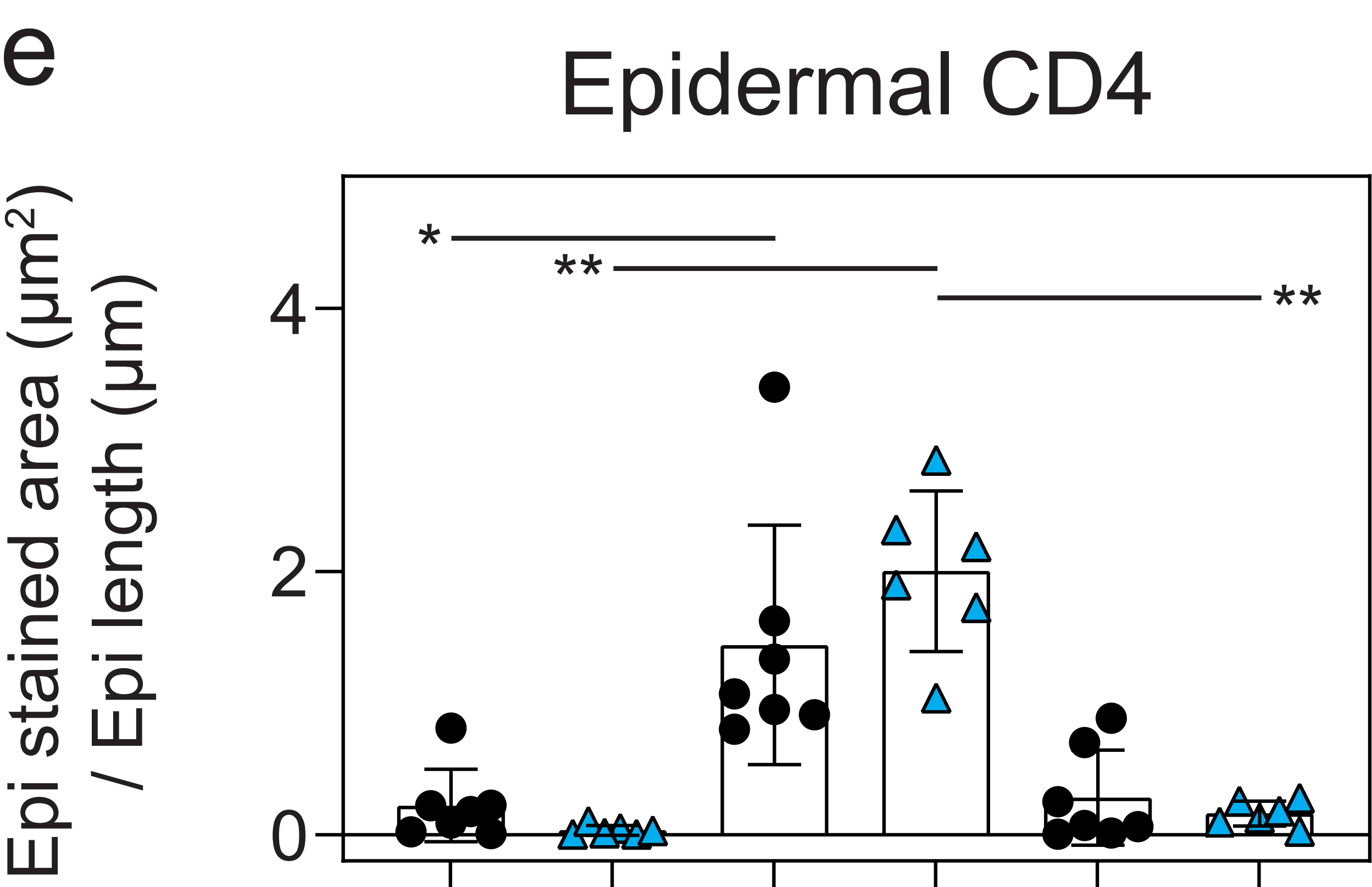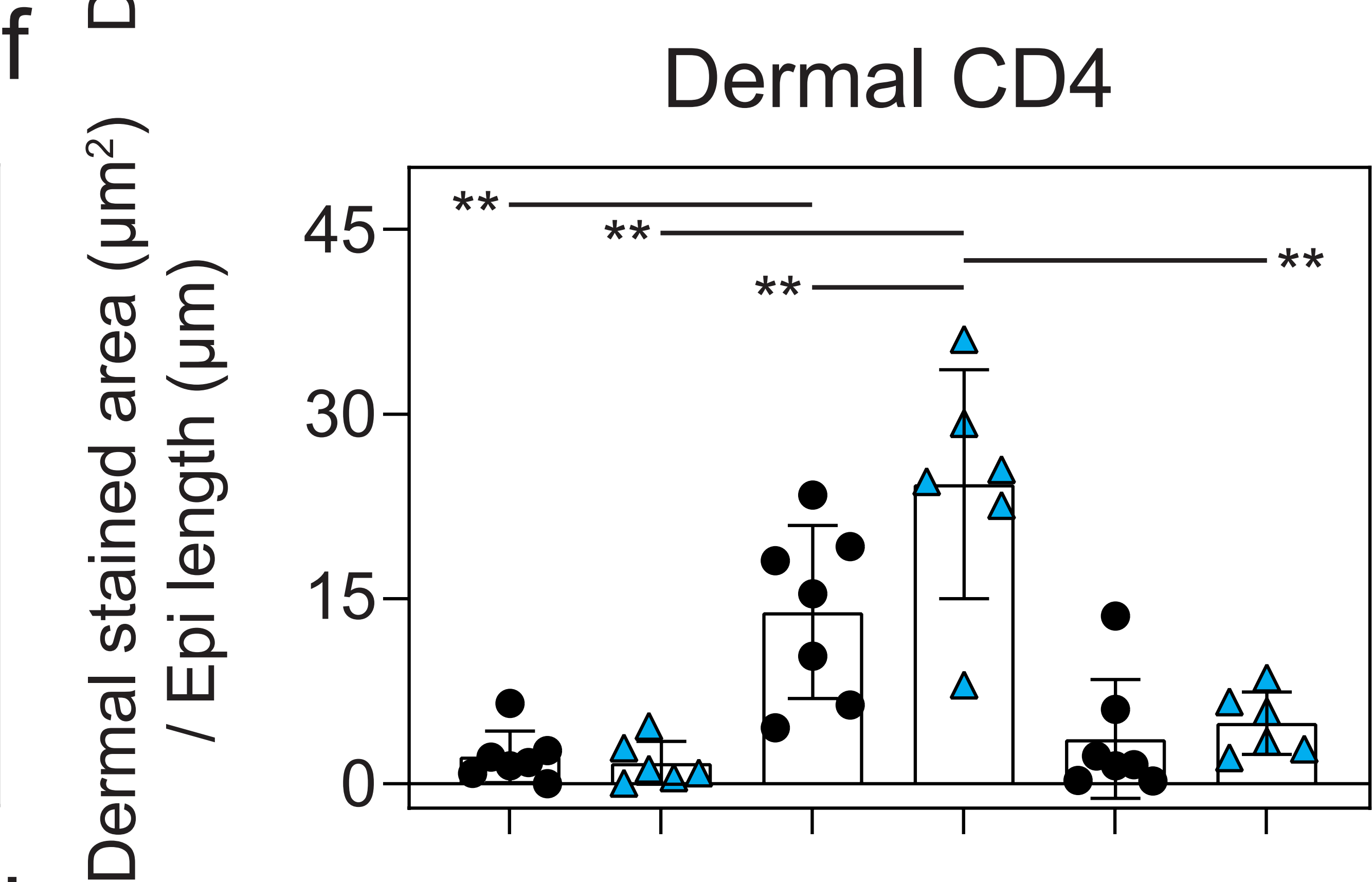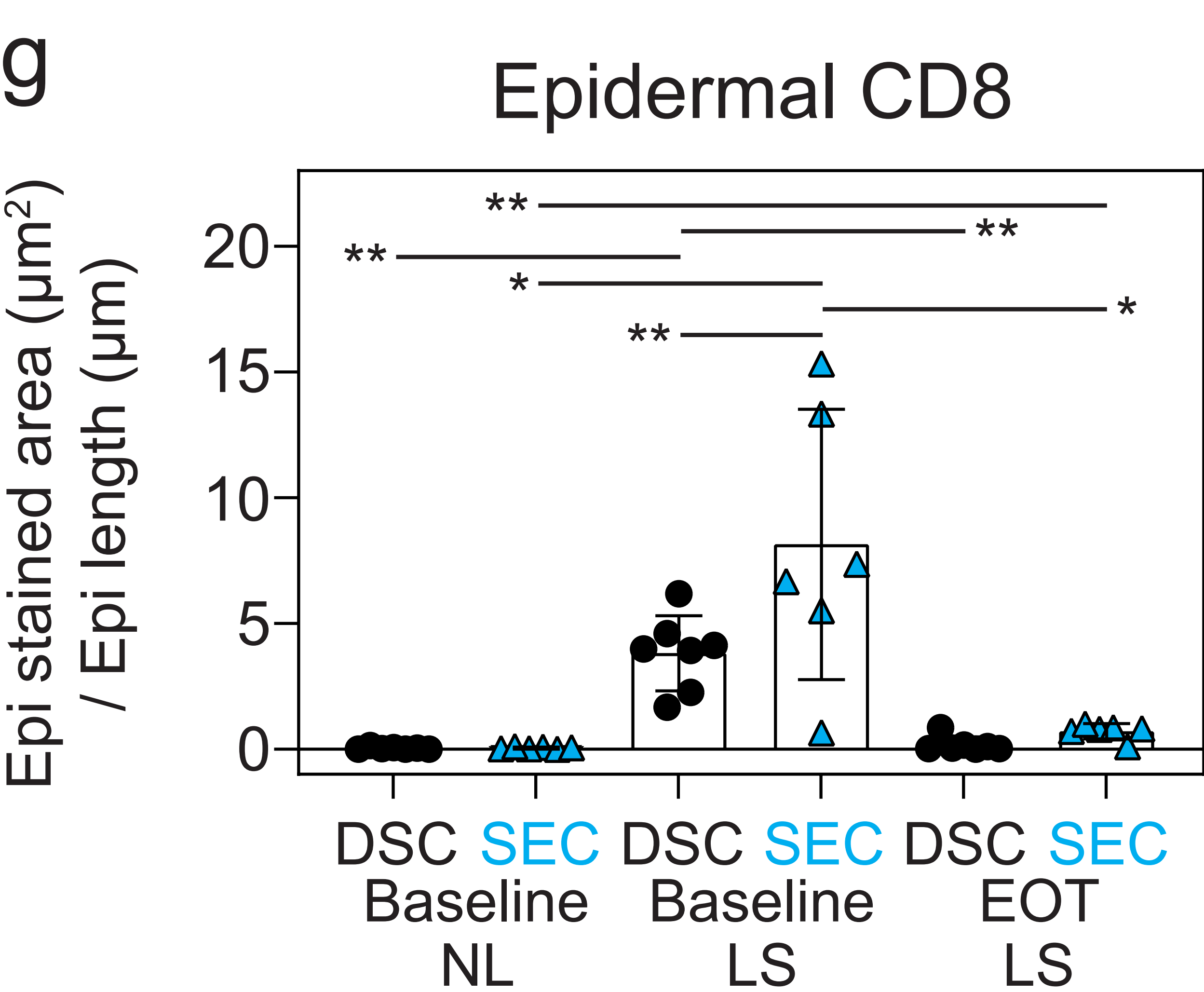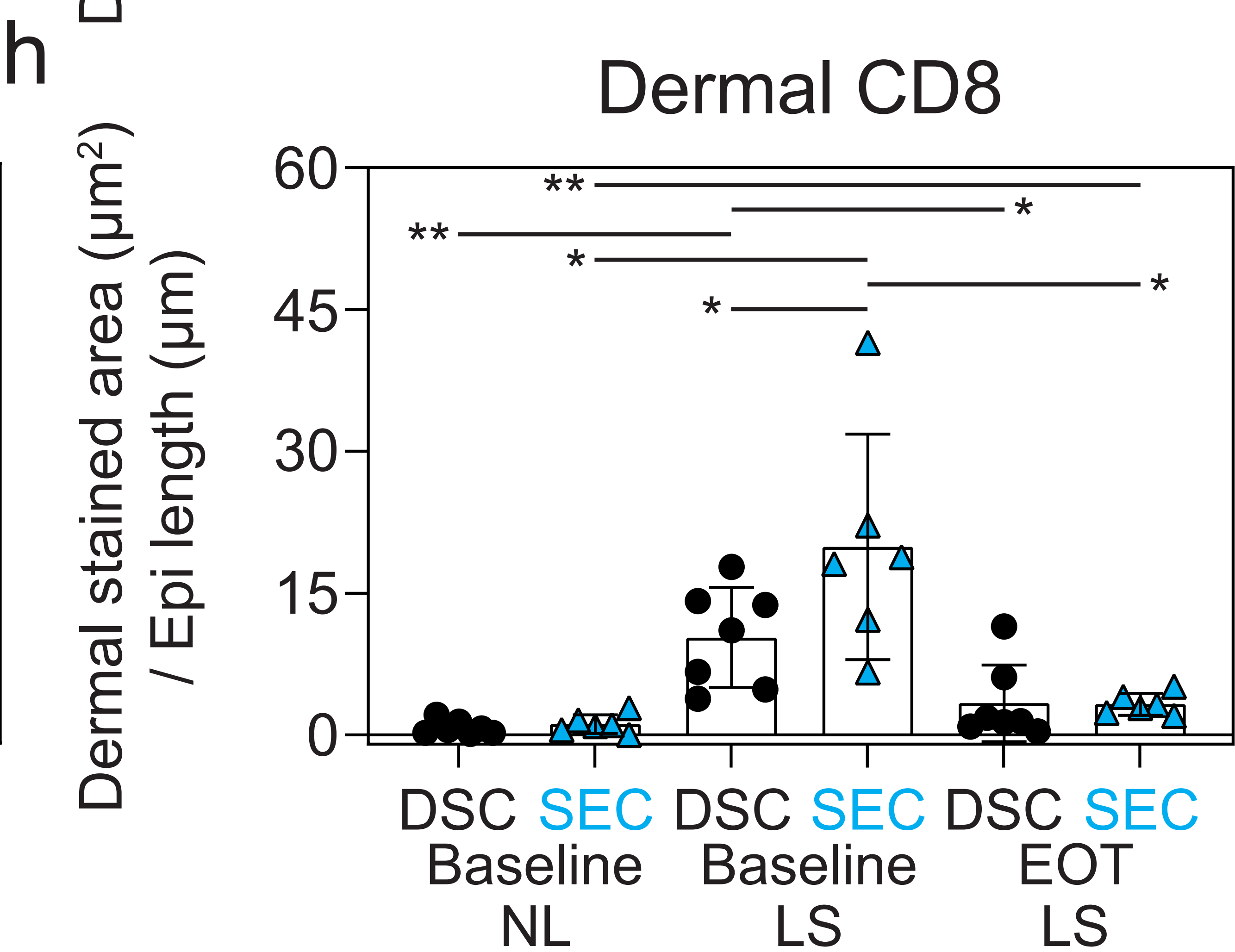

Supplement: Supplementary file 1 [file ijms-25-06086-s001.zip › figure_s1.pdf]

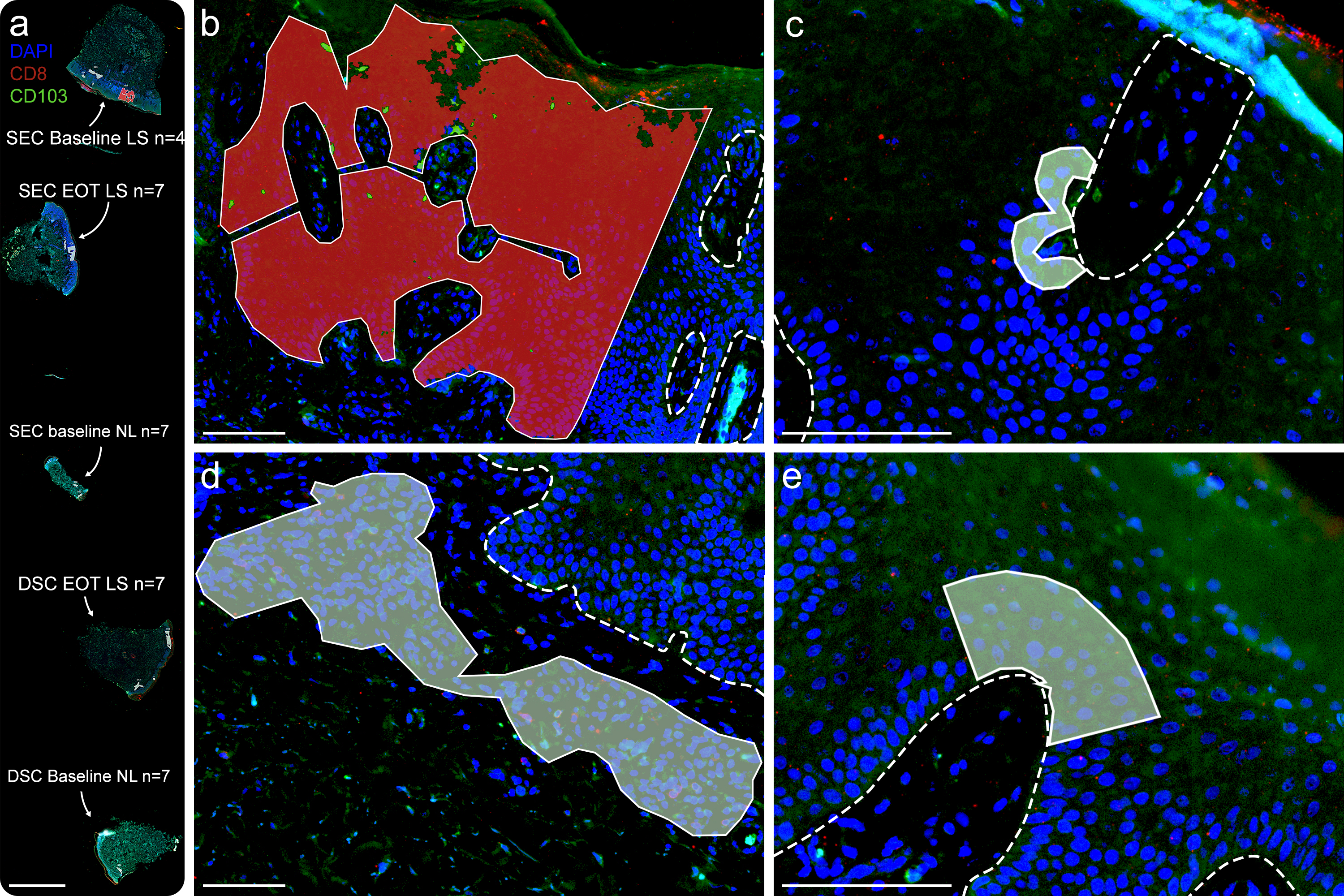

Supplement: Supplementary file 1 [file ijms-25-06086-s001.zip › figure_s12.jpg]

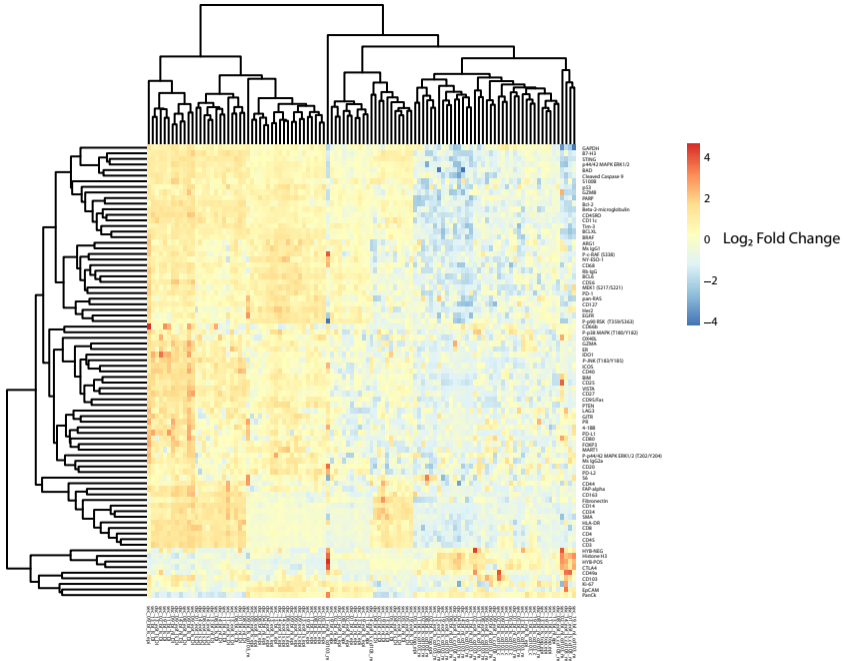

Supplement: Supplementary file 1 [file ijms-25-06086-s001.zip › figure_s13.pdf]

## SEC - CD103<sup>+</sup> cell microenvironment

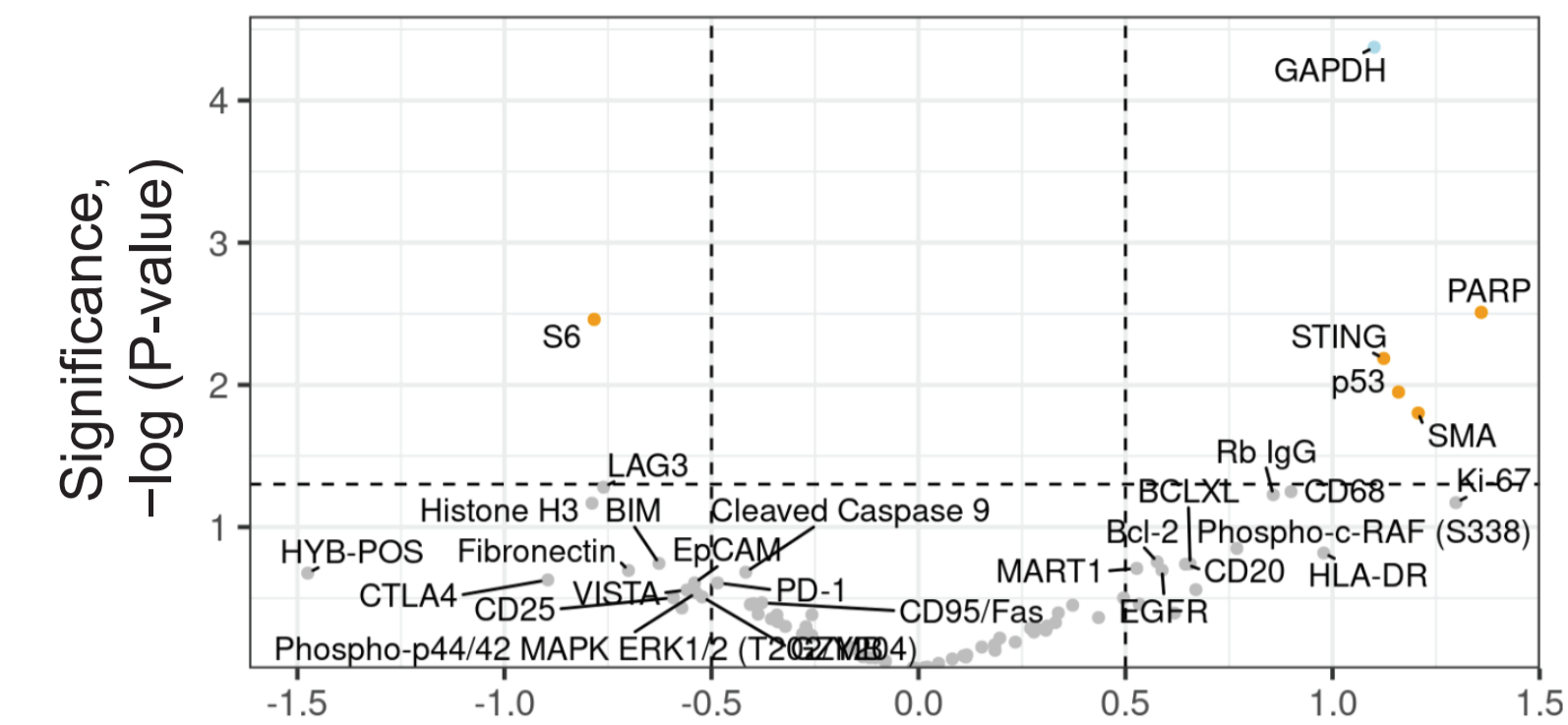

Supplement: Supplementary file 1 [file ijms-25-06086-s001.zip › figure_s14.pdf]

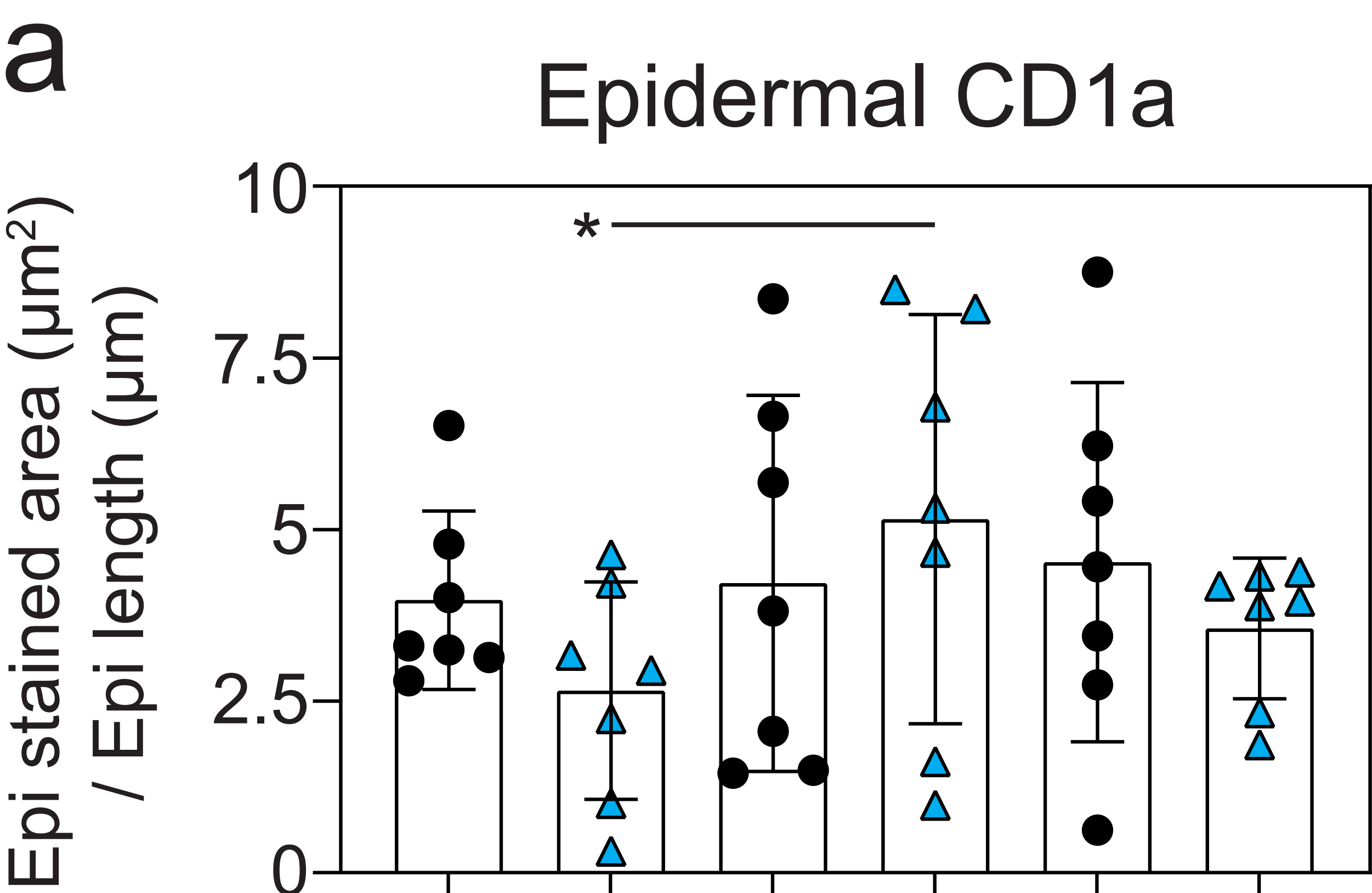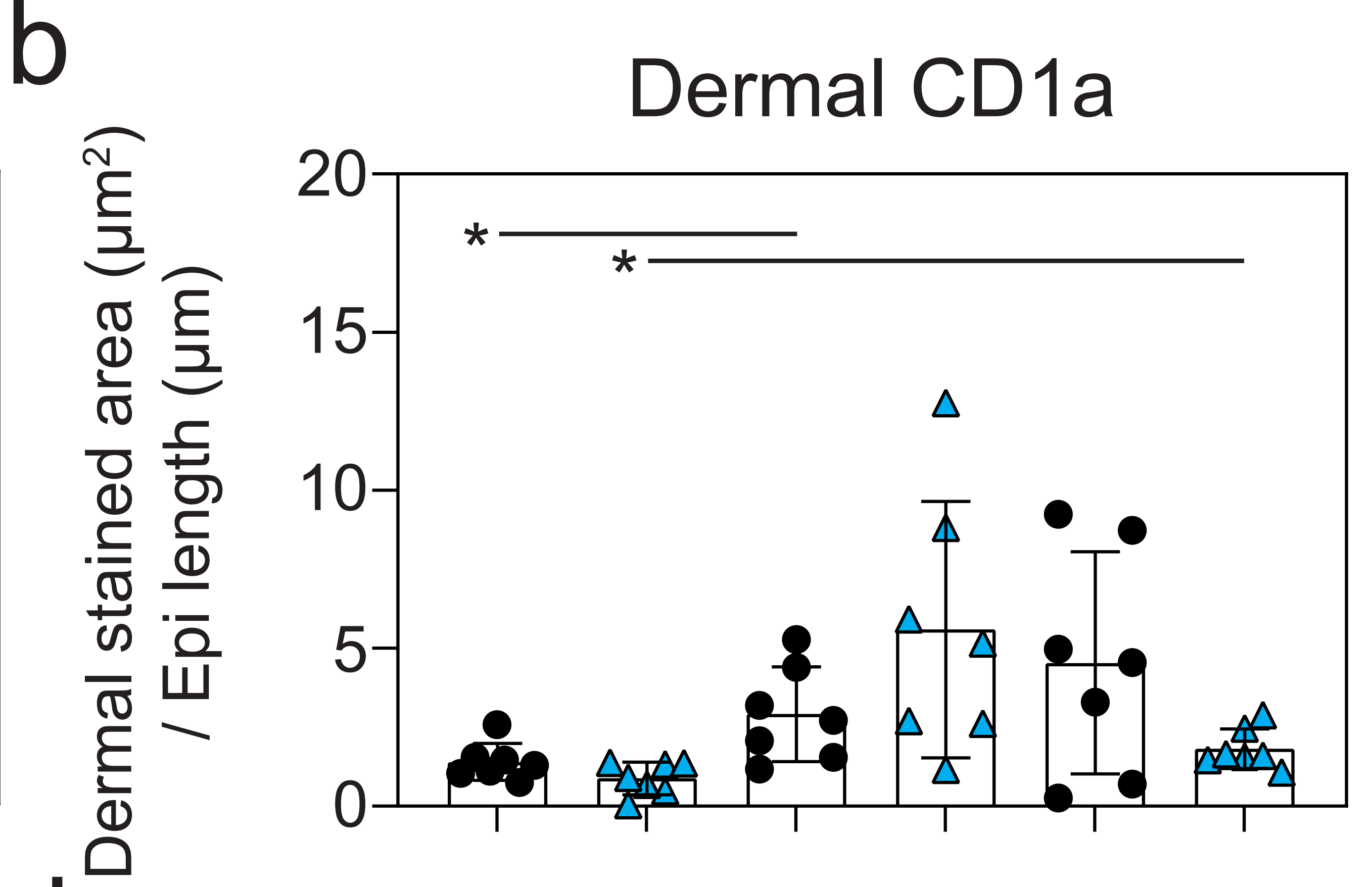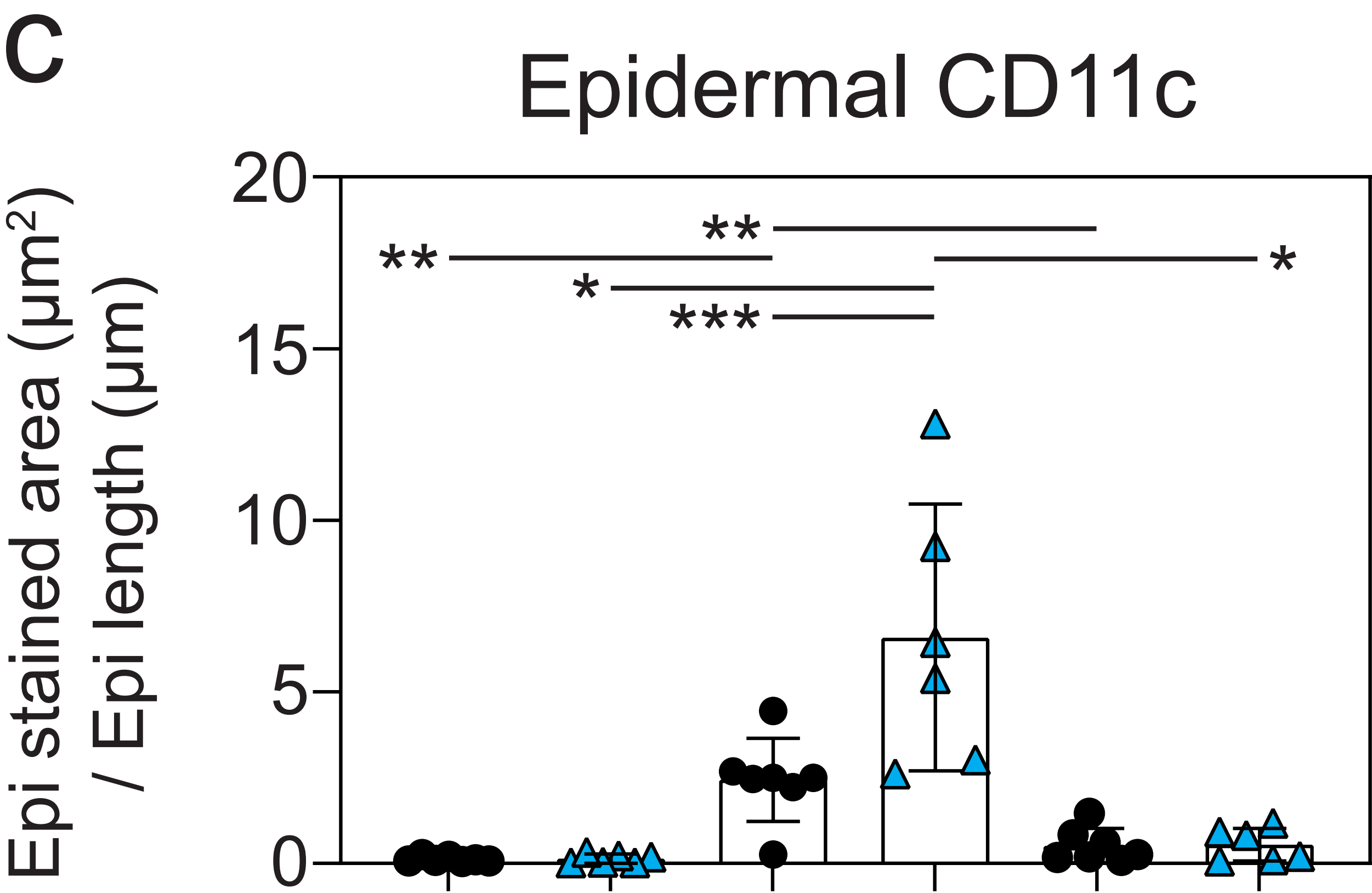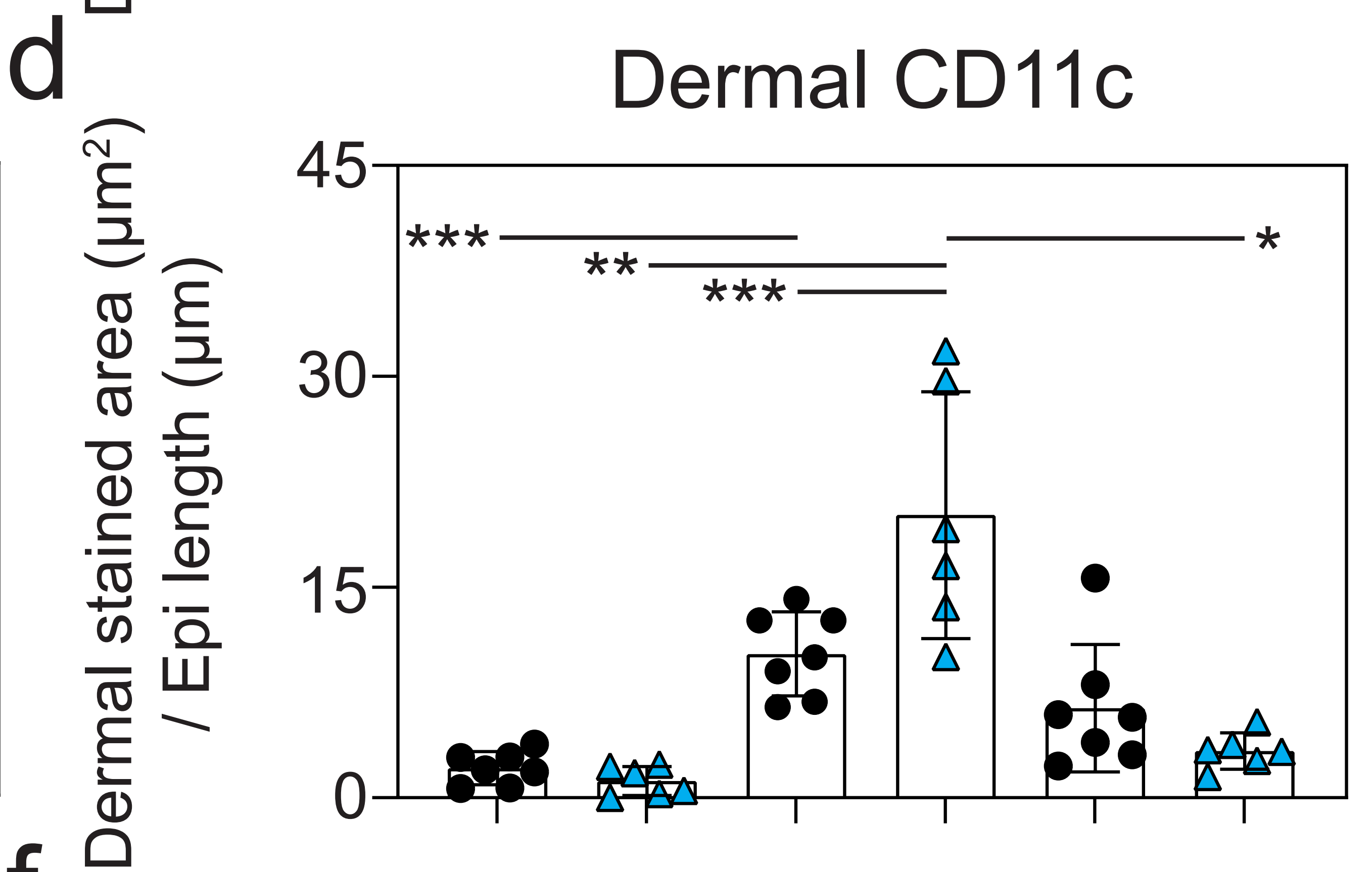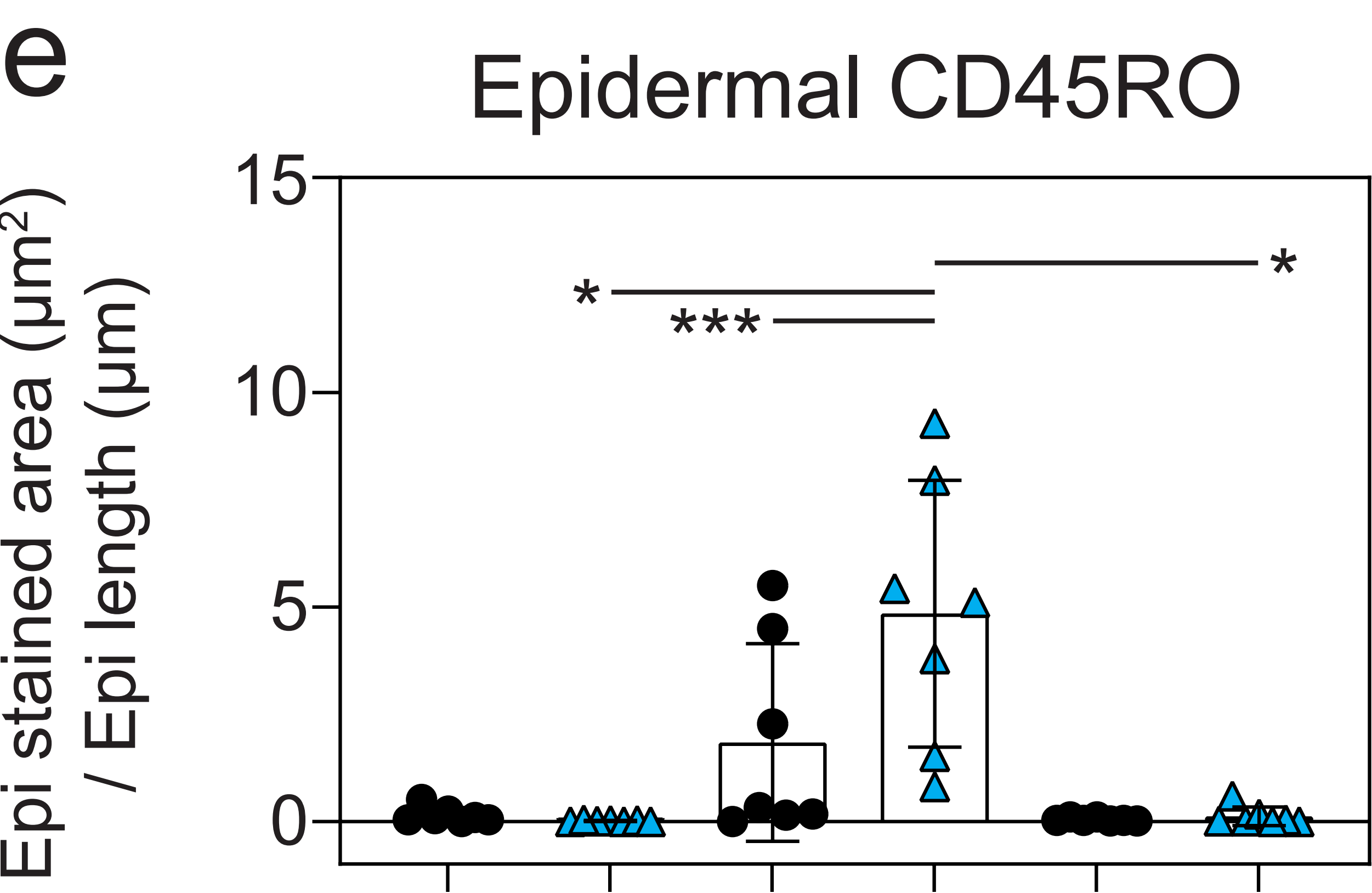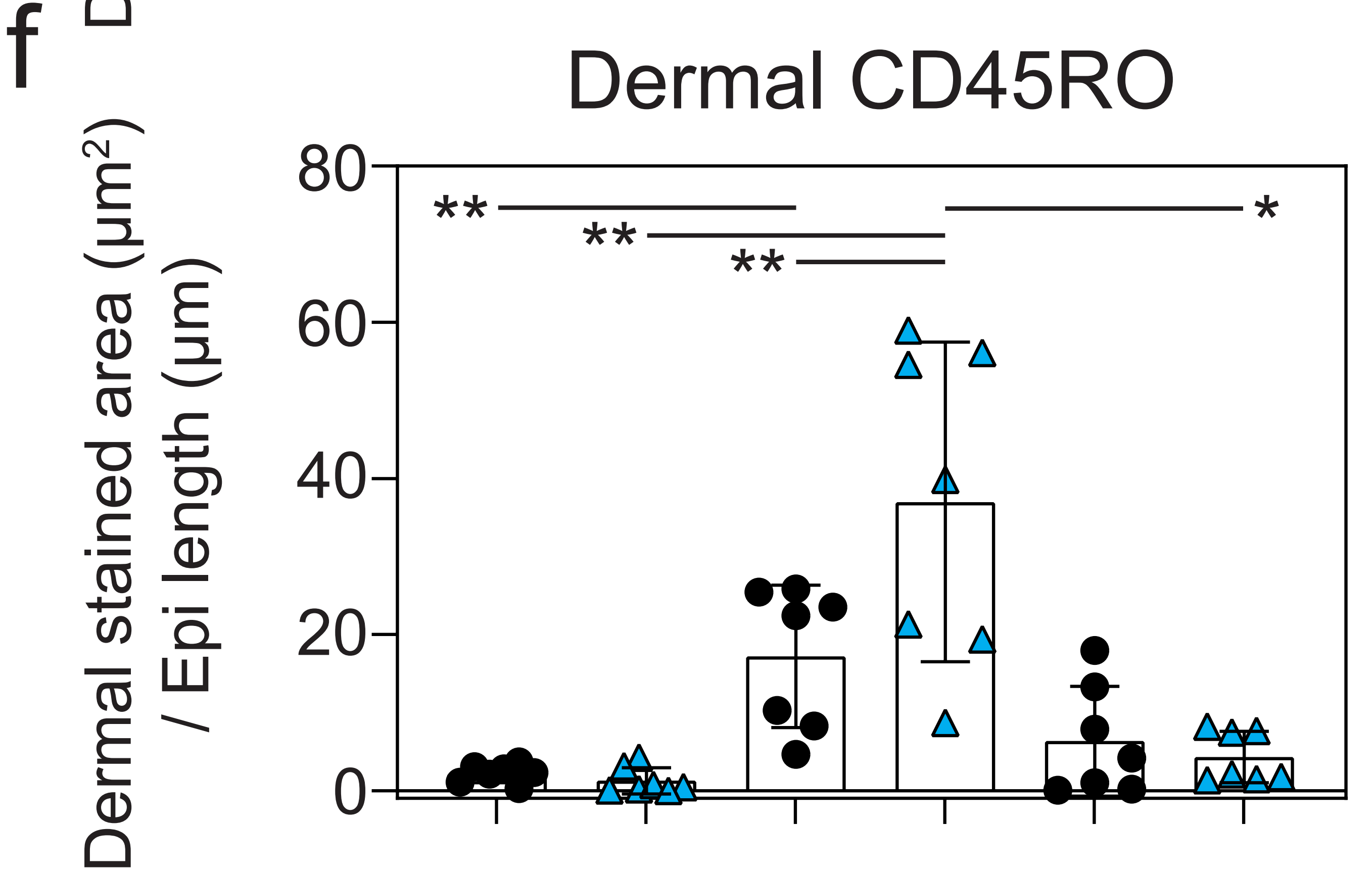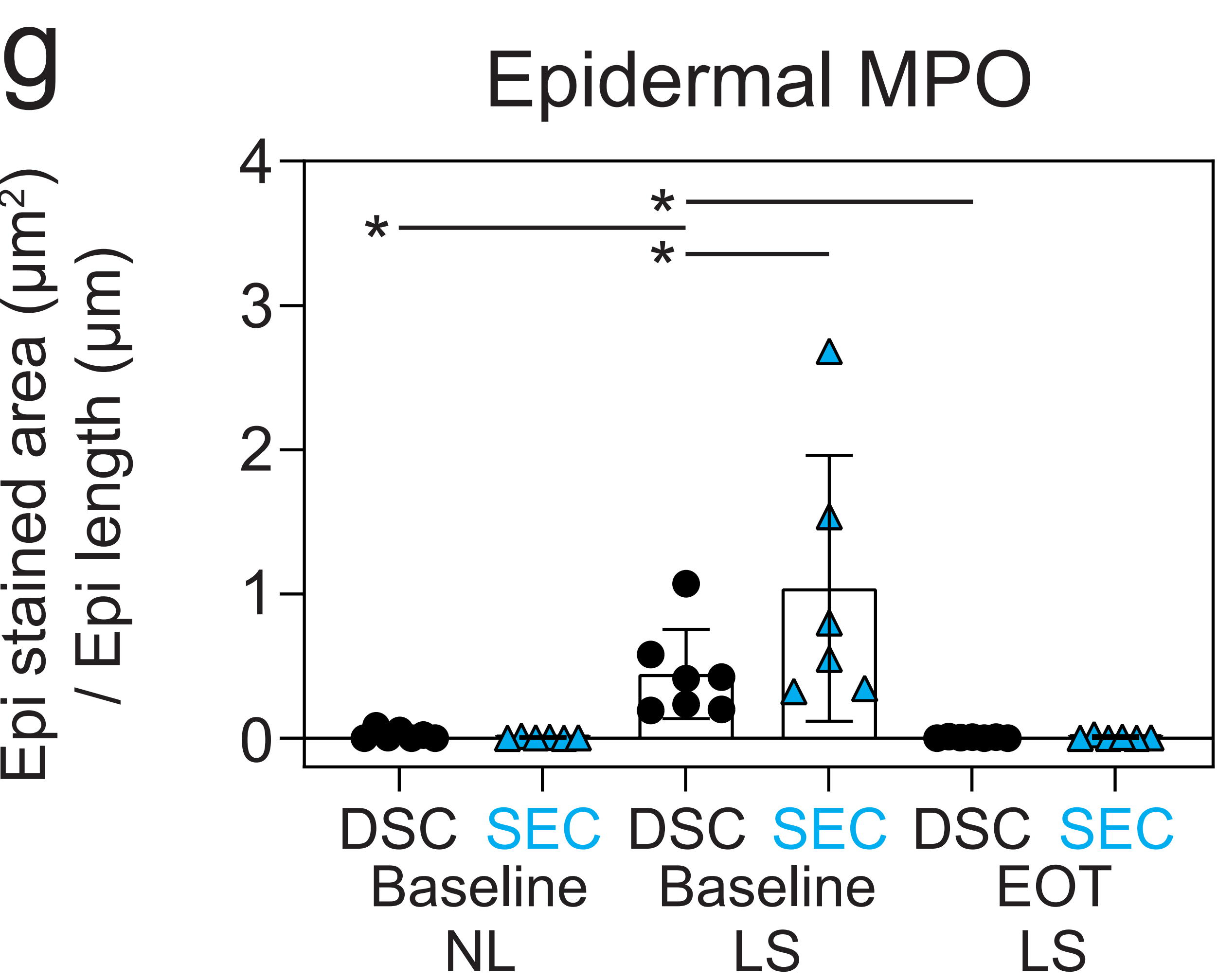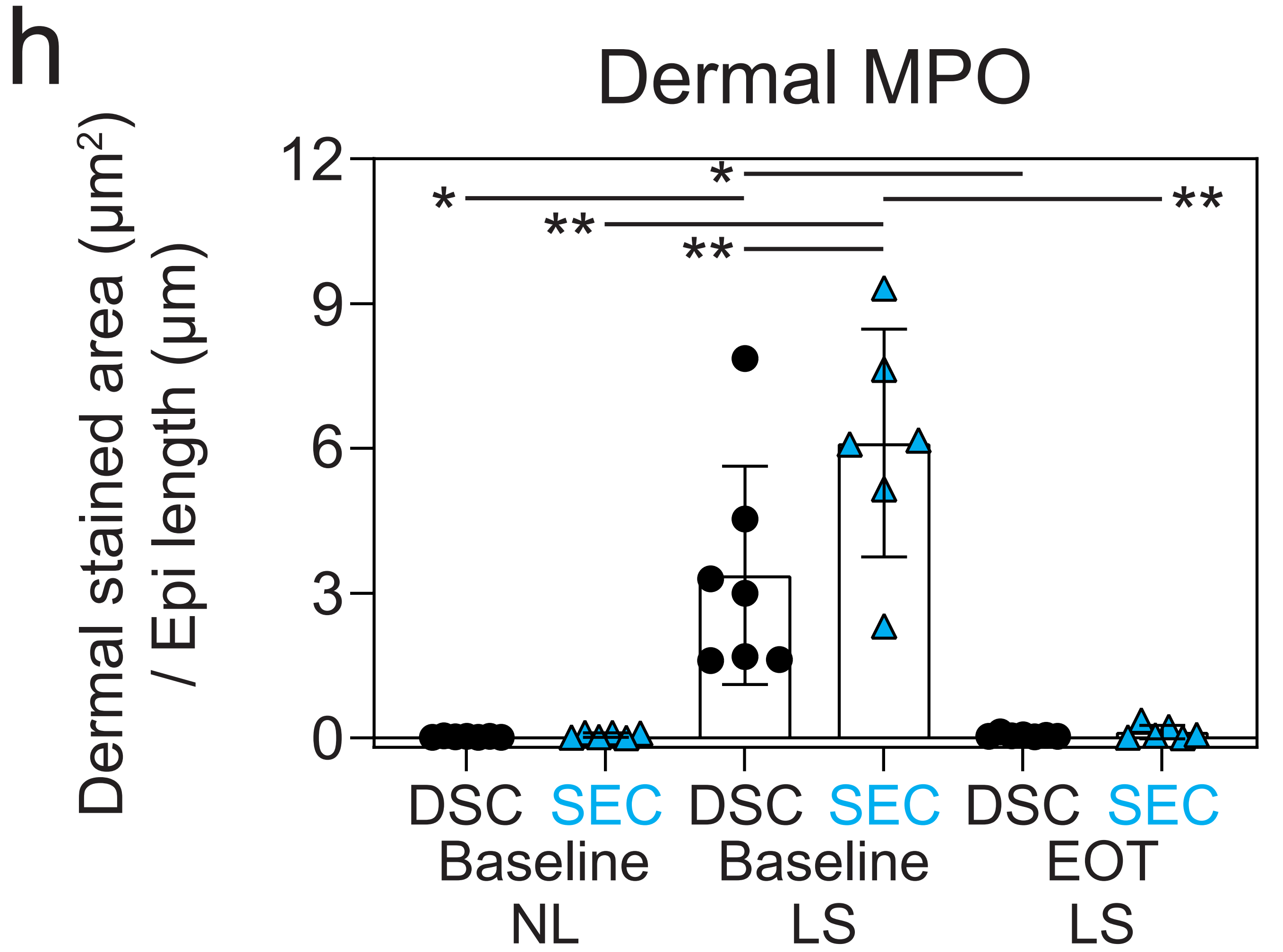

Supplement: Supplementary file 1 [file ijms-25-06086-s001.zip › figure_s3.pdf]

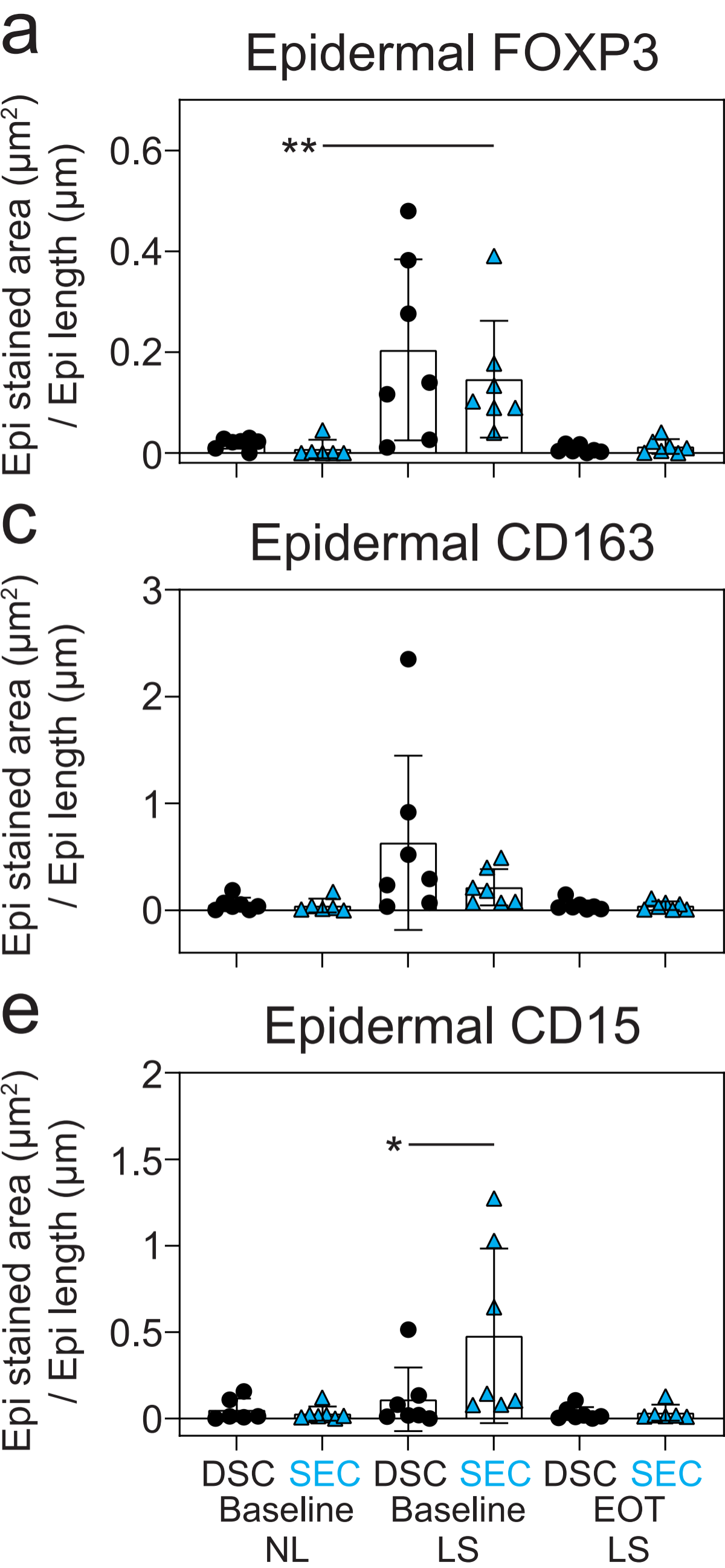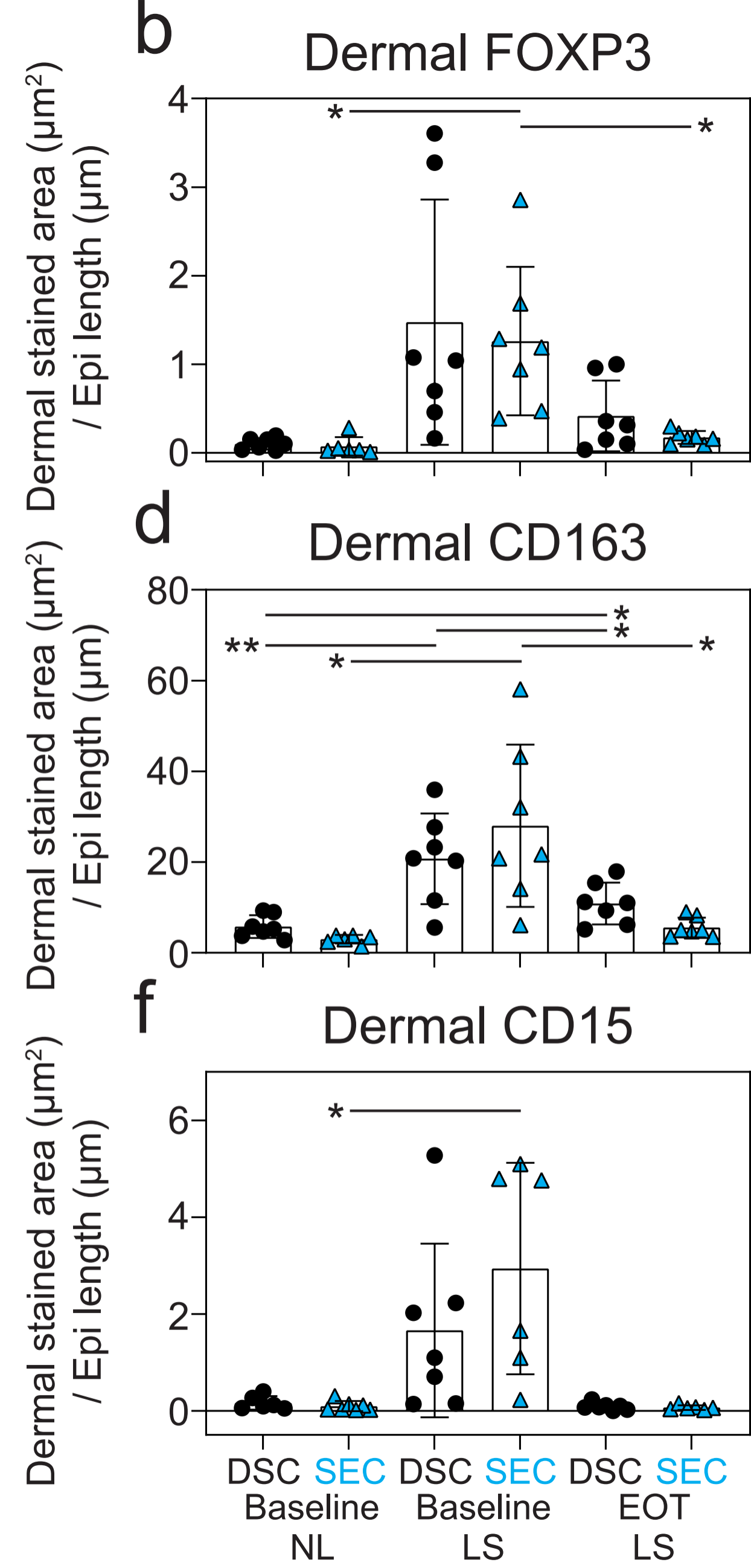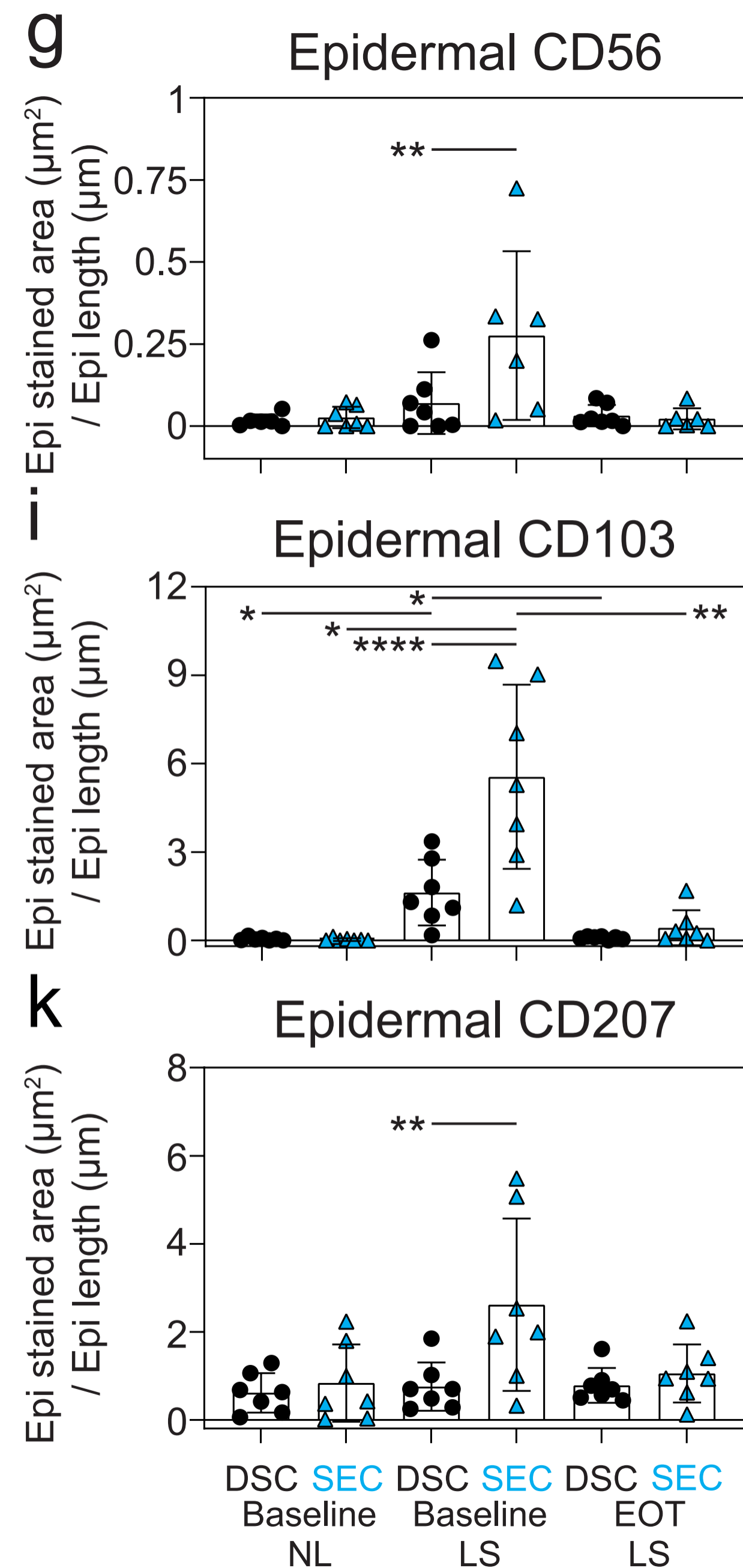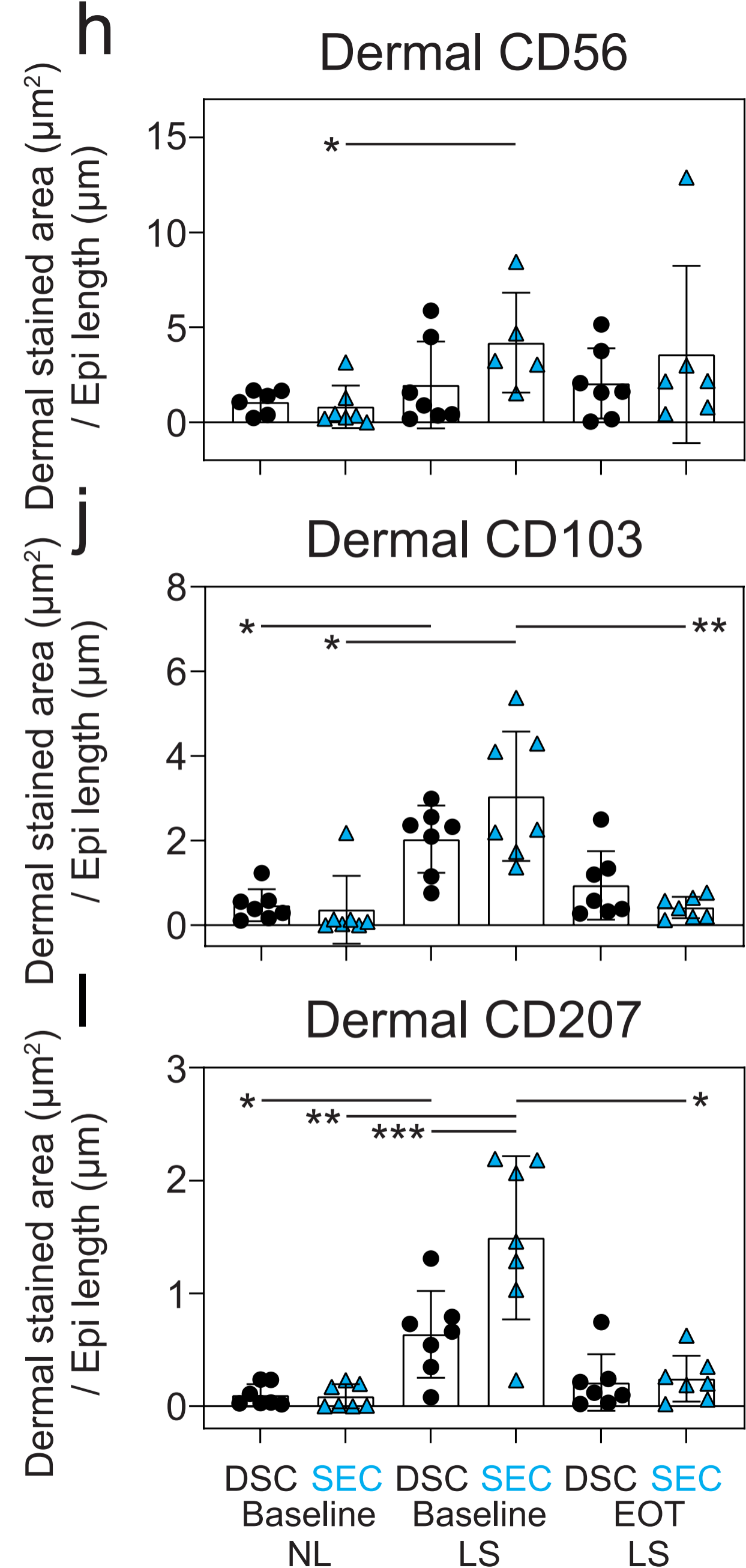

Supplement: Supplementary file 1 [file ijms-25-06086-s001.zip › figure_s4.pdf]

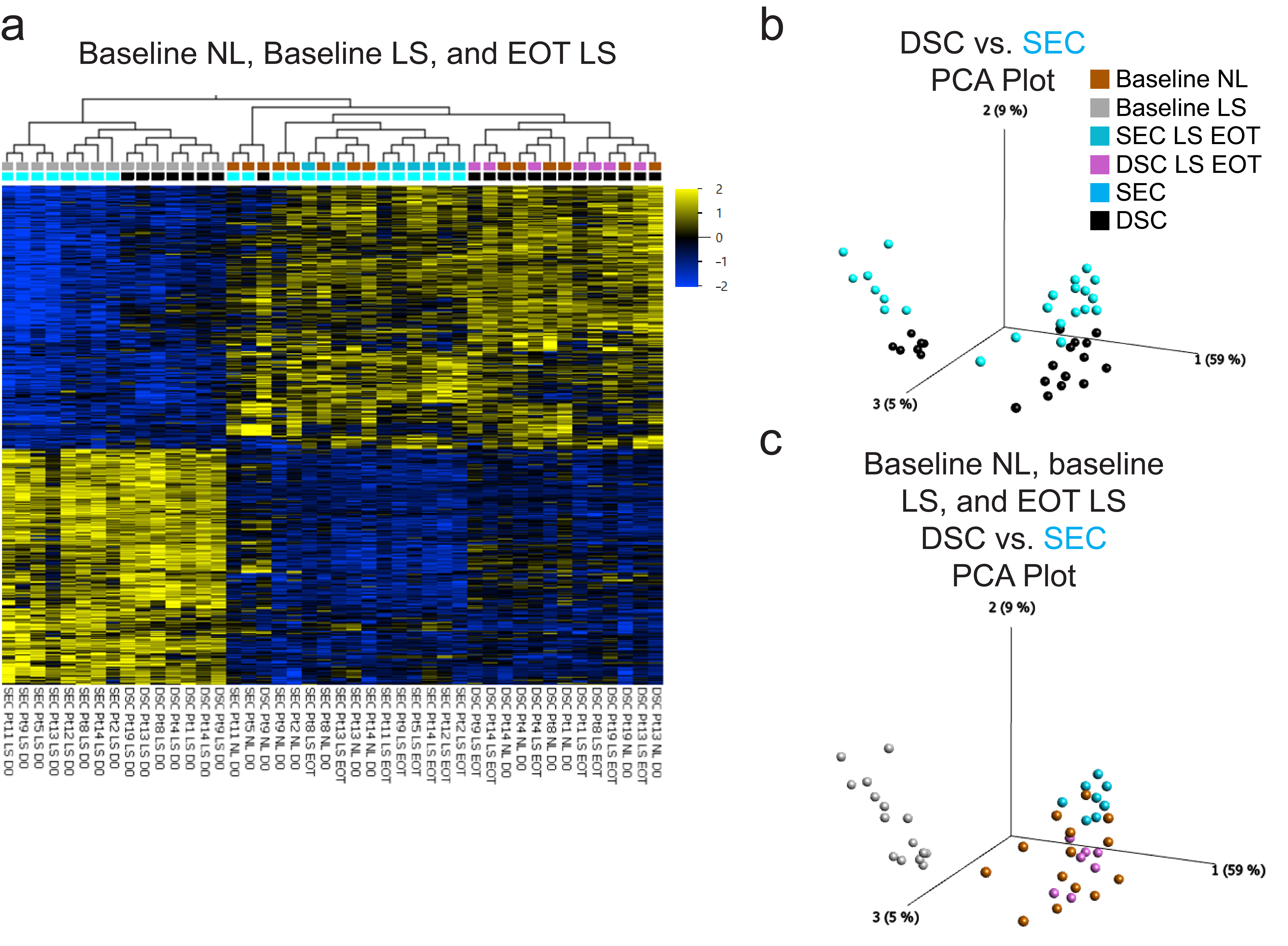

Supplement: Supplementary file 1 [file ijms-25-06086-s001.zip › figure_s5.jpg]
